# Ras Effector Mutant Expression Suggest a Negative Regulator Inhibits Lung Tumor Formation

**DOI:** 10.1371/journal.pone.0084745

**Published:** 2014-01-28

**Authors:** Guillaume Vandal, Benjamin Geiling, David Dankort

**Affiliations:** Department of Biology, McGill University, Montréal, Quebec, Canada; Children's Hospital Boston, United States of America

## Abstract

Lung cancer is currently the most deadly malignancy in industrialized countries and accounts for 18% of all cancer-related deaths worldwide. Over 70% of patients with non-small cell lung cancer (NSCLC) are diagnosed at a late stage, with a 5-year survival below 10%. KRAS and the EGFR are frequently mutated in NSCLC and while targeted therapies for patients with EGFR mutations exist, oncogenic KRAS is thus far not druggable. KRAS activates multiple signalling pathways, including the PI3K/Akt pathway, the Raf-Mek-Erk pathway and the RalGDS/Ral pathway. Lung-specific expression of BrafV600E, the most prevalent BRAF mutation found in human tumors, results in Raf-Mek-Erk pathway activation and in the formation of benign adenomas that undergo widespread senescence in a Cre-activated Braf mouse model (Braf^CA^). However, oncogenic KRAS expression in mice induces adenocarcinomas, suggesting additional KRAS-activated pathways cooperate with sustained RAF-MEK-ERK signalling to bypass the oncogene-induced senescence proliferation arrest.

To determine which KRAS effectors were responsible for tumor progression, we created four effector domain mutants (S35, G37, E38 and C40) in G12V-activated KRAS and expressed these alone or with BrafV600E in mouse lungs… The S35 and E38 mutants bind to Raf proteins but not PI3K or RalGDS; the G37 mutant binds to RalGDS and not Raf or PI3K and the C40 mutant is specific to PI3K. We designed lentiviral vectors to code for Cre recombinase along with KRAS mutants (V12, V12/S35, V12/G37, V12/E38 or V12/C40) or EGFP as a negative control.. These lentiviruses were used to infect *Braf^CA^* and wild-type mice. Surprisingly there was a significant decrease in tumor number and penetrance with each KRAS effector domain mutant relative to controls, suggesting that KRAS directly activates effectors with tumor suppressive functions.

## Introduction

Lung cancer is the leading cause of cancer deaths worldwide [1] and can be categorized into two main histological subtypes: small cell lung cancer and non-small cell lung cancer (NSCLC). The latter can be further divided into three subtypes: large cell carcinoma, squamous cell carcinoma and adenocarcinoma. In never smokers, 62% of lung cancers diagnosed are of the adenocarcinoma subtype, which makes it the most frequent subtype among that group, while it accounts for 19% of smoking-induced lung cancers [2]. Over 70% of NSCLC patients present at an advanced stage of the disease and have a poor 5-year survival (7–9% survival at stage IIIb and 2% at stage IV) [3]–[5].

Multiple activating mutations in oncogenes have been found in lung adenocarcinomas, including in the following genes: *EGFR* (39%), *KRAS* (20%), *ALK* fusions (notably with *EML4*) (4%), *ERBB2* (3%) and *BRAF* (3%), which are found to be mutually exclusive [6]–[8]. Interest in targeted therapies has been increasing in recent years after sensitivity to treatment with Erlotinib and Gefitinib, EGFR tyrosine kinase inhibitors, was correlated to the *EGFR* mutational status [9], [10]. Recently, Crizotinib, a RTK inhibitor specific for the ALK and Met receptors [11], has been approved by the US Food and Drug Administration as a new targeted therapy against *ALK*-rearranged NSCLC [12], [13]. Unfortunately, not only are NSCLC patients with *KRAS* mutations not responsive to targeted therapies against *EGFR* or *ALK*, *KRAS* mutations are also predictive of a reduced survival under conventional chemotherapy when compared to patients with *EGFR* mutations [14]–[16].

The *RAS* family of genes is composed of three members with high sequence homology: *HRAS*, *NRAS* and *KRAS*, of which there are two alternative splicing isoforms, *KRAS 4A* and *4B*. In lung adenocarcinoma, *KRAS* is the predominant *RAS* gene found mutated (20%), whereas *HRAS* and *NRAS* are found mutated in other types of epithelial cancers [17]. The protein products of *KRAS* (KRAS 4A and KRAS 4B) are small GTPases of 21 kDa that serve as molecular switches for signal transduction from the cellular membrane, by alternating between an active, GTP-bound state and an inactive GDP-bound conformation [18], [19]. Oncogenic *KRAS* mutations in humans almost exclusively alter codons 12 (85% of *RAS* mutations), 13 (14%) or 61 (1.5%) [7] and disrupt the catalytic activity of KRAS, leaving RAS in a permanent active, GTP-bound “active” state [20], [21]. GTP-bound RAS proteins adopt a conformation that exposes two regions named switch I (residues 32–40, also known as the effector domain), and the switch II region (residues 60–76) [22]. The switch I and II regions allow Ras to activate downstream signalling by recruiting Ras effector proteins to the plasma membrane. These Ras effector proteins interact with Ras through one of three types of Ras binding domains (RBD). The first type is found on the phosphatidylinositol 3-kinase (PI3K) p110 subunits (α, β, γ or δ) [23], [24]. The second interaction domain is a Raf-type RBD that is found in Raf proteins and also in Tiam1 [25]. The third type of RBD is the RA domain (RalGDS/AF-6 or Ras-association domain), found in RalGDFs (Ral-guanine nucleotide dissociation stimulators also known as RalGEFs, Ral-guanine exchange factors) [26], [27]. It is widely held that this interaction with effector proteins and/or membrane proximity can alter catalytic activity and/or substrate partner availability thereby facilitating signal transduction. There is a series of RAS effector domain mutants (RAS^ED^ mutants), which allow for the differential activation of downstream signalling pathways [24], [28]. For instance, expression of point activated HRAS^V12^ bearing an S35 or E38 mutation preferentially interacts with and activates Raf proteins with minimal recruitment of PI3K and RalGDS members [24], [28]. In a similar fashion C40 RAS^ED^ mutants preferentially activate the PI3K pathway and G37 RAS^ED^ mutants induce predominantly activate Ral-GDS. *T*he KRAS effector domain mutants have an abrogated binding to certain effectors (S35 and E38 bind to Raf proteins but not RalGDS or PI3K; G37 binds to RalGDS but not Raf or PI3K; C40 binds to PI3K but not Raf and RalGDS) [28]–[30] That stated, there are a number of other proteins that interact with RAS's effector domain in a GTP-dependent manner, which are thus potential Ras effector proteins [23]. The binding of these proteins to different RAS^ED^ mutants has been assessed allowing the use of RAS^ED^ mutants to probe RAS signalling further [23].

A number of mouse models exist to explore the role RAS plays in tumor initiation and progression [31], [32]. Two genetically engineered mouse (GEM) models have been developed to express physiological levels of KRas^G12^ mutants following Cre-mediated recombination: *KRas^LSL^* mice, where recombination leads to KRas^G12D^ expression [33] and *KRas^V12-IRES-βGeo^* mice, which express KRas^G12V^ following Cre expression [34]. Lung specific [33] or systemic activation [34] of these alleles leads to the formation of atypical adenomatous hyperplasia and epithelial hyperplasia of the bronchioles as early as 2 weeks post-infection. These lesions progress to adenomas and eventually to adenocarcinomas [33], [34]. To determine whether Ras-Raf-Mek-Erk signalling was sufficient to initiate this cancer phenotype, the *Braf* locus was engineered to express constitutively active Braf^V600E^ protein at physiological levels following Cre-mediated recombination in *Braf^CA^* mice [35]. Lung-specific Braf^V600E^ expression causes the formation of atypical adenomatous hyperplasia and multiple tumors with an adenomatous morphology. At the earlier stages, these tumors are phenotypically similar to those observed with the *KRas^LSL^* mice [33], [35]. Tumors that develop in both models stain positive for the type II pneumocyte antigen, SP-C, and are negative for the Clara cell marker, CC10. While many of the KRas^G12V^-driven lung tumors remain adenomas due to a stable proliferative arrest (i.e.: oncogene-induced senescence) [36], adenocarcinomas appear as early as 16 weeks post-induction. Strikingly Braf^V600E^ lung adenoma progression to adenocarcinomas is a very rare event and has not been observed before 40 weeks post- Braf^V600E^ expression. Much like Kras-induced adenomas [36], these Braf^V600E^ lung adenomas appear to be senescent [35]. Together this suggests that while sustained RAF-MEK-ERK MAPK pathway plays a role as an initiator of disease [35], [37], [38], additional RAS effector proteins mediate a signal required for lung adenoma progression.

We sought to use genetic complementation in mouse lungs to determine whether an additional Kras effector protein(s) could cooperate with constitutive MAPK pathway activation to bypass oncogene-induced senescence (OIS) and permit tumor progression. Specifically we expressed different G12V-activated KRAS effector domain mutants (KRAS^V12^, KRAS^V12/S35^, KRAS^V12/G37^, KRAS^V12/E38^ or KRAS^V12/C40^) [23], [24], [28]–[30] alone or in conjunction with induction of Braf^V600E^ expression in *Braf^CA^* mice and assessed tumor formation and pathology. To do this we designed a Gateway-compatible lentiviral vector (gLEX-iCL) to express a cDNA (here either EGFP or KRAS mutants) along with bicistronic expression of the Cre recombinase (here to permit Braf^V600E^ expression). These lentiviruses were used to infect the lungs of wild-type and *Braf^CA/+^* mice to assess how each effector domain mutant of KRAS cooperates with sustained MAPK pathway activation. Tumor size was significantly elevated in Braf^V600E^ lesions expressing either KRAS^V12^ or KRAS^V12/C40^, suggesting co-activation of the PI3K pathway cooperate to increase tumor growth. Surprisingly we found that, expression of each activated KRAS^ED^ lead to a substantial decrease in Braf^V600E^ induced tumors relative to the EGFP control suggesting the existence of a KRAS activated negative regulator of tumorigenesis.

## Results

### Development and titration of lentiviral expression vectors

**Figure 1:**
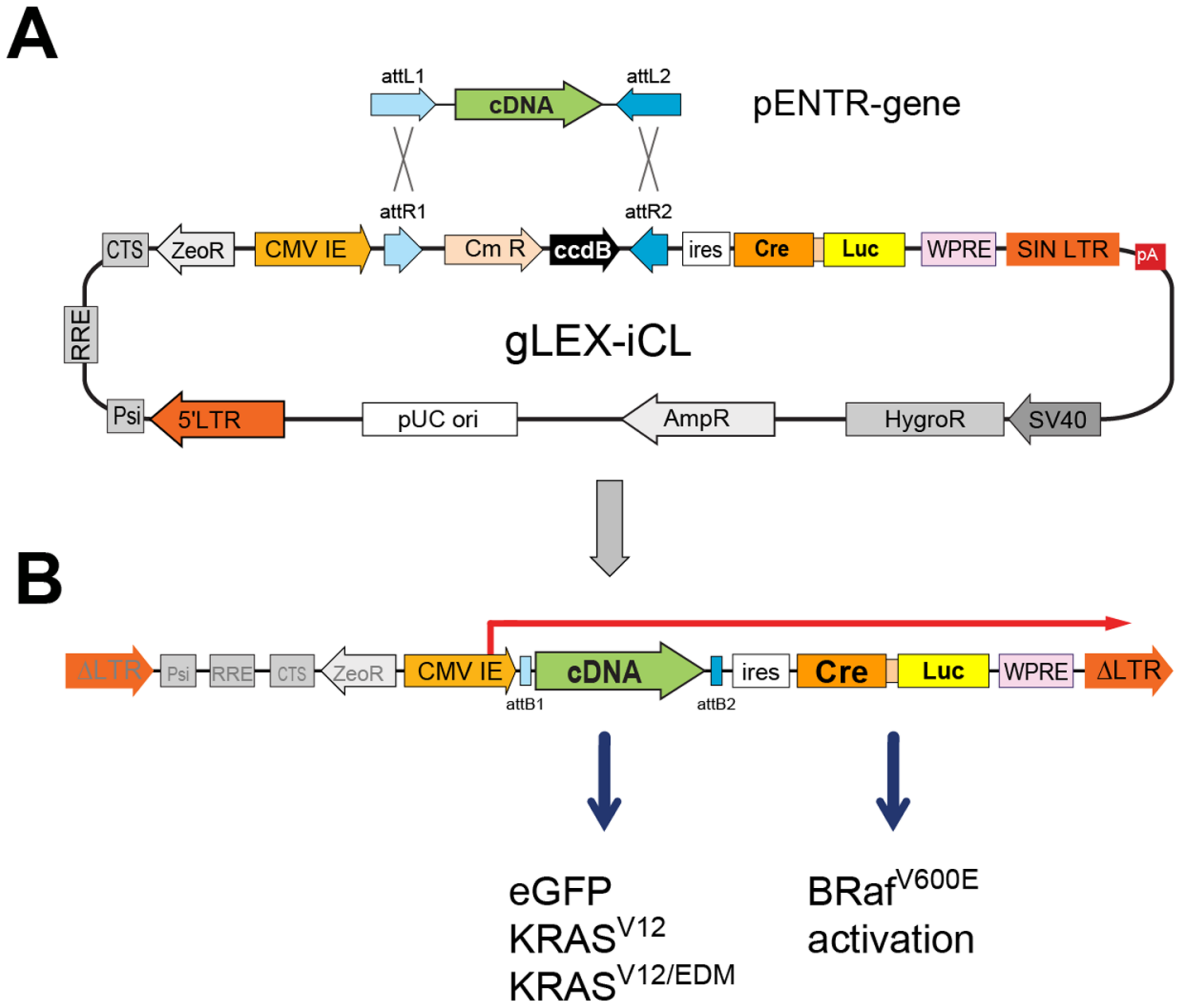
Plasmids used for lentivirus production and subsequent mouse infections/Lentiviral vectors. **A**) Schematic of a generalized two-plasmid LR recombination reaction between a generalize entry vector containing a cDNA and gLEX-iCL, a Gateway compatible lentivirus encoding Cre(T2A)Luc **B**) The resulting recombinant lentiviral expression vector when integrated contains a single CMV-driven bicistronic transcript encoding a cDNA (EGFP, KRasG12, or effector domain mutants) and downstream of an ires, Cre(2a)Luc fusion to induce Braf^V600E^ expression in *Braf^CA^* mice.

To efficiently co-express activated KRAS, KRAS^ED^ and EGFP along with Cre recombinase we engineered a bicistronic lentiviral vector, gLEX-iCL (Figure 1). This second-generation lentiviral vector contains a Gateway selection cassette to facilitate the cloning of cDNAs upstream of an internal ribosome entry sequence (ires), which allows for the translation of the downstream Cre recombinase and luciferase. The latter proteins are encoded as one open reading frame and are separated by a *Thosea asigna* virus-derived 2A peptide allowing for “translational cleavage” [39], [40] between the Cre and luciferase proteins [41]. Lentiviral expression vectors containing the KRAS effector domain mutants or EGFP as a control were produced via Gateway recombination. The selective activity of the KRAS effector domain mutants and the Cre recombinase activity were confirmed *in vitro* (supplemental data, Figure S1, S2, S3).

**Figure 2:**
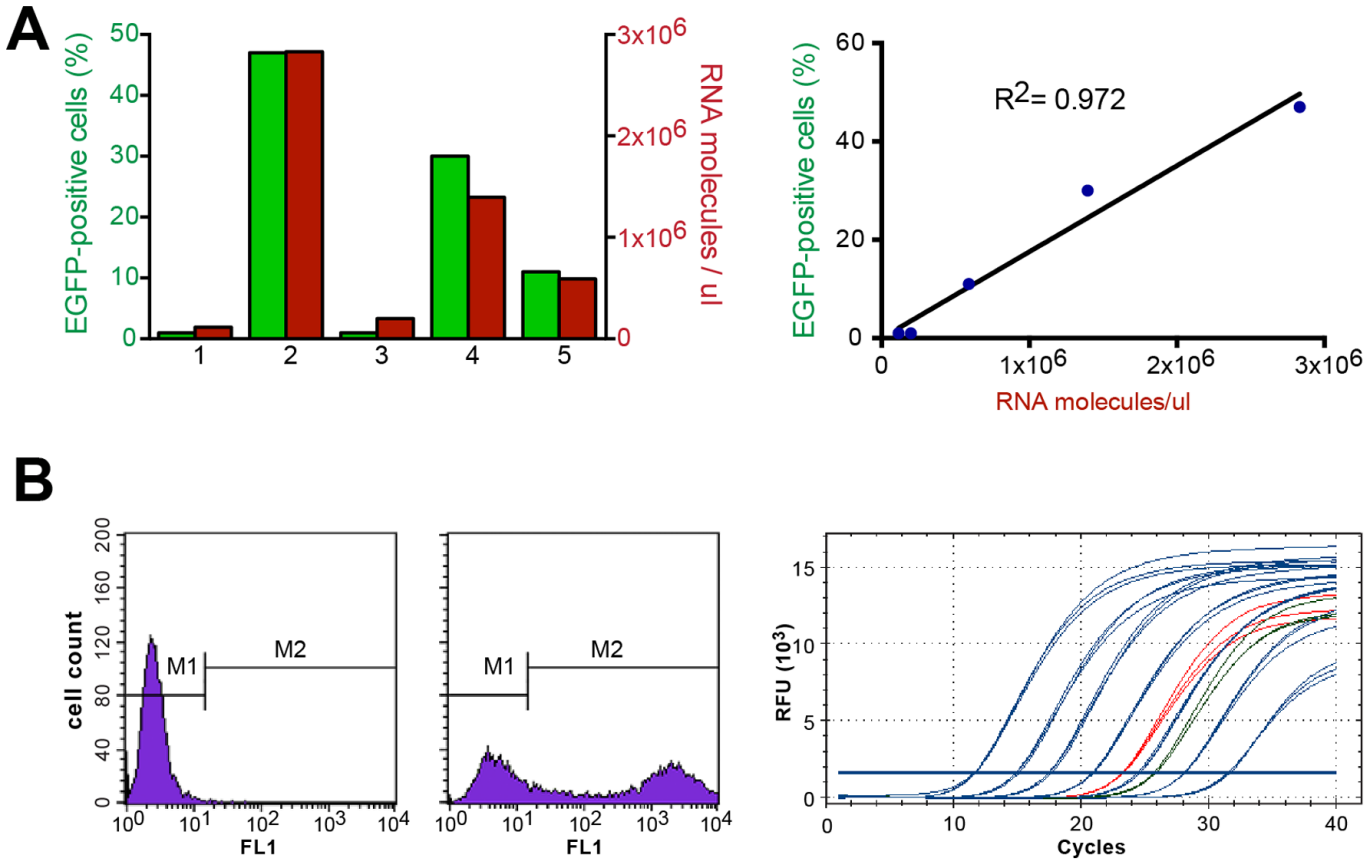
Titration of lentiviruses. **A**) Correlation between the number of viral RNA molecules from five lentiviruses from different vectors (1. pLEX-EGFP-iCL, 2. pLEX-EGFP-iPuro, 3 pLEG-EGFP-iCL-shRNA(p53), 4. pLEG-EGFP- iPuro, 5. pLEG-EGFP-iPuro-shRNA(p53), all coding for the EGFP protein, and the FACS analysis of 293T cells for EGFP-positive cells that were infected with those same viruses. **B**) 2×10^5^ 293T cells were infected with 1/10 and 1/100 dilutions of previously concentrated virus (left, 1/10 dilution) or no virus (middle). Right: quantitative PCR analysis after RNA extraction and reverse transcription of the 1/10 and 1/100 dilutions of the EGFP-iCL virus. Blue curves: standard DNA ranging from 10 fg/µl to 10 ng/µl in 10-fold increments; red curve: 1/10 dilution of EGFP-iCL; green curve: 1/100 dilution. **C**) Correlation between the number of infectious particles (y axis) and the number of cDNA molecules obtained after viral RNA extraction, reverse transcription and qPCR (x axis). Squares and circles represent different LEX-EGFP-iCL viral preparations and each IU/µl value was obtained by infecting 293T cells with two different volumes of virus.

Lentiviruses, to be used as vectors, are often titered using clonogenic assays for drug resistance markers, flow cytometric analysis for fluorescent protein markers or with immune-based assays for viral or encoded proteins engineered into the virus. The LEX-iCL viruses encoding KRAS derivatives lack a selectable marker to determine their titre directly, thus an alternate method of lentivirus titration was needed, which would be applicable to all lentiviruses used for mouse infections. As such, we used reverse transcription quantitative PCR (RT-qPCR) to measure the amount of lentiviral vector genomic RNA in different virus productions. To this end we initially focused our efforts on LEX-EGFP-iCL, where we could correlate the number of RNA molecules with the number of infectious units as determined by flow cytometric analysis for EGFP in this vector. The primers used for the RT-qPCR analysis are specific for the Woodchuck Hepatitis Virus Posttranscriptional Regulatory Element (WPRE) region. This element is commonly used in lentiviral vectors to enhance expression, thus rendering the RT-qPCR titration method applicable to any lentivirus containing this WPRE region [42], [43]. Unconcentrated lentiviral supernatants were divided into 2 aliquots. RNA was extracted from one viral aliquot and the number of virion RNA molecules was determined by RT-qPCR. The titre of lentivirus from the other aliquot was determined by infecting HEK 293T cells and subsequently determining the number of EGFP-positive cells using fluorescence-activated cell sorting (FACS). The number of virion RNA molecules was strongly correlated (r^2^ = 0.972) to the percentage of EGFP-positive cells for the five different undiluted lentiviruses of varying sizes (Figure 2A). These findings are consistent with others [42], [43] and demonstrate that RT-qPCR of RNA isolated from lentiviral supernatant can be used as a surrogate to measure viral titre.

We additionally determined the feasibility of this approach to quantify concentrated virus. Specifically we generated seven independent lentiviruses preparations of LEX-EGFP-iCL (EGFP-iCL) and using serial dilutions of these concentrated viruses we determined titre with FACS relative to number of RNA molecules following the same qRT-PCR approach. Despite ultracentrifugation and concentration of the virus by over 200-fold we obtained similar results (supplemental data, Figure S4). Hence, the titre of all lentiviruses produced in the same manner can be determined by measuring the number of RNA molecules through RT-qPCR. We noted that the absolute slope of this line was affected by alterations to the protocol used for the production and concentration of lentiviruses. Specifically during the optimization of large-scale virus production and concentration we noted that multiple factors may modify the ratio of infectious to defective virion particles (e.g. virus collection time, pH of media)(data not shown). As such, all large-scale lentiviral production followed the specific protocol described in materials and methods.

### Lentiviral transduction to initiate Braf^V600E^ expression

To determine whether lentiviruses containing Cre recombinase could be administered efficiently to the lungs of mice, we use intranasal administration of LEX-EGFP-iCL, a virus that encodes both EGFP and Cre recombinase. *Braf^CA/+^* or their wild type littermates were infected with 10^8^ infectious units (IU) of LEX-EGFP-iCL. The mice were pretreated with sodium caprate prior to the intranasal instillation of the virus to increase infection efficiency as this has been shown to increase the viral transduction through disruption of the tight junctions [44]. Using this method and 10^8^ IUs of LEX-EGFP-iCL virus, we observed tumors formed in each mouse with an average of 33 tumors/per mouse when assessed at 16 weeks post-infection (Table 1). The tumors initiated with lentiviral Cre in *Braf^CA^* mice had a papillary adenomatous phenotype indistinguishable from those formed with Adenovirus Cre [35]. These adenomas stain positive for the type II pneumocyte marker surfactant protein C (SP-C) and negative for the Clara cell antigen 10 ([35] and supplemental data, Figure S5). Moreover, we did not detect tumors in any of the wild-type mice infected with LEX-EGFP-iCL (n = 8) demonstrating that lentiviral integration and Cre recombinase activity do not themselves initiate tumor formation.

**Table 1:** Tumor penetrance, expressed in average number of tumors observed per lung ± SEM, using the intranasal instillation technique of *Braf^CA/+^* mice.

	8weeks		16weeks	
Virus	Mice with tumors	Avg. tumors/lung	Mice with tumors	Avg. tumors/lung
EGFP	3/3	1.0	5/5	33.0±13.2
KRAS^V12^	5/5	1.2±0.2	5/5	2.2±0.6
KRAS^V12/S35^	1/3	1.0	3/4	2.7±1.2
KRAS^V12/G37^	1/3	1.0	2/3	1.0
KRAS^V12/E38^	1/3	1.0	3/4	2.0±1.0
KRAS^V12/C40^	2/5	1.0	3/6	1.0

Using the same viral dose we then infected *Braf^CA/+^* and wild type littermate mice with lentiviruses additionally encoding KRAS^V12^ or the activated effector domain mutant KRAS^S35^, KRAS^G37^, KRAS^E38^, KRAS^C40^ by intranasal instillation. Surprisingly, while each mouse infected with LEX-iCL virus expressing KRas^V12^ developed tumors by 16 weeks postinfection, there was a 15-fold reduction in tumor number relative to *Braf^Ca^* activation alone (Table 1). Additionally, a large variance in tumor formation in LEX-EGFP-iCL mice was apparent with tumor number ranging from 1 to 79 tumors per animal. We believe that this variation, in part, is due to the intranasal administration of sodium caprate. Caprate at these concentrations is viscous and this pretreatment appeared to render breathing while under sedation more difficult. This irregular breathing made the subsequent administration of the virus variable. Moreover, the large variation and relatively low number of tumors formed with intranasal lentivirus administration led us to explore other means of administering virus. We then compared intranasal versus direct intratracheal administration of dye and found that a much higher proportion of dye enters the lungs when infection intratracheally (not shown). We thus chose to conduct subsequent experiments with intratracheal virus administration.

**Figure 3:**
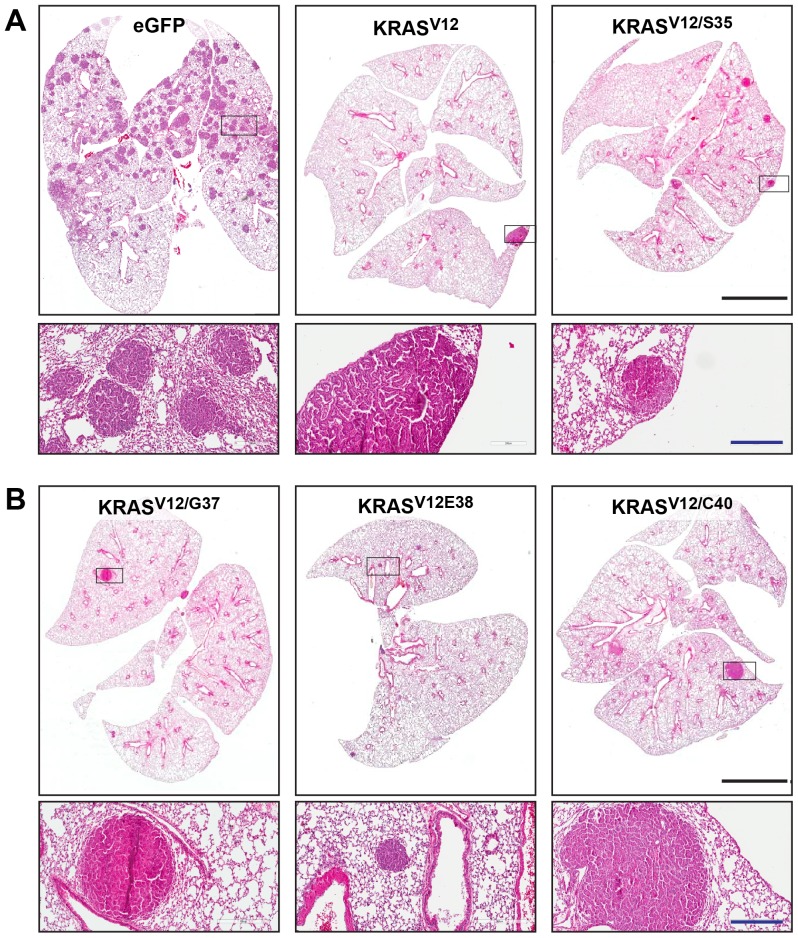
KRas expression inhibits tumor formation in after Cre-mediated expression of Braf^V600E^ in *Braf^CA^* ^/+^ mice. Sections of lungs from *Braf^CA/+^* mice infected with LEX-iCL lentiviruses expressing EGFP, KRAS^V12^ or the indicated KRAS^V12/ED^ mutants. Slides were stained with haematoxylin and eosin and are shown at low (upper panels) and high (lower panels) magnification. The box in the upper panels depicts region shown at higher power. All images are representative for each lentiviral construct. Bars, 3 mm.

Intratracheal administration of 10^8^ IUs of LEX-EGFP-iCL was sufficient to initiate over 1000 tumors in *Braf^CA^* mice as early at the 8- and 16- week time points. In this instance caprate pretreatment caused a transient reduction in breathing rate, which returned to normal prior to lentivirus administration (see methods). Additionally, there was a decreased variability in tumor number by this method. Again the lung sections of *Braf^CA/+^* mice receiving EGFP-iCL virus contained many well-differentiated adenomas per section (Figure 3). Using EGFP-iCL or similar vectors we reproducibly obtained 1000 lung adenomas in each mouse (Table 2 and not shown).

**Table 2:** Tumor penetrance, expressed in average number of tumors observed per lung ± SEM, in *Braf^CA/+^* mice infected by tracheal intubation with 1–2×10^8^ infectious units of indicated lentivirus.

	8weeks		16weeks	
Virus	Mice with tumors	Avg. tumors/lung	Mice with tumors	Avg. tumors/lung
EGFP	2/2	>1000	4/4	>1000
KRAS^V12^	1/2	2.0	1/2	1.0
KRAS^V12/S35^	1/2	3.0	2/2	8.0±7.0
KRAS^V12/G37^	0/2	0	2/3	3.5±1.5
KRAS^V12/E38^	2/3	2.5±0.5	3/3	17.0±5.8
KRAS^V12/C40^	1/2	2.0	2/3	2.0

### Expression of KRAS^ED^ inhibits Braf^V600E^-driven tumor initiation

Having determined that we could routinely infect *Braf^CA/+^* mice and obtain a large number of tumors, we infected *Braf^CA/+^* mice with LEX-KRAS^V12^-iCL. KRAS^V12^ coexpression resulted in a large decrease in tumor number relative to the control EGFP-iCL virus both at high (10^8^ IU, Table 1) and lower viral titres (10^7^ IU, supplementary Table S1). Histological analysis of the tumors (n = 24) induced in each of these conditions revealed that all KRAS^V12^-induced tumors possessed a papillary adenoma phenotype (Figure 3). Surprisingly, there was a lack of adenocarcinoma in *Braf^CA/+^* mice induced with lentiviruses expressing KRAS^V12^ and Cre. Presumably the lack of adenocarcinomas is reflective of a low tumor number. Indeed, in *Kras^LSL^* mice expressing KRas^G12D^ only a fraction of tumors progress to adenocarcinoma [45]. To determine the effects of KRAS^V12^ on its own, we infected wild type littermate control mice with lentiviruses at high titre (10^8^ IU) using the tracheal intubation method. We observed a reduced penetrance (2 of 5 mice developed tumors) and these mice developed only 2 and 4 tumors per mice.

To determine if any of the effector mutants genetically cooperate with Braf^V600E^ to induce adenocarcinoma formation, we used intratracheal administration of lentiviruses to express each of the KRAS^ED^ mutants. In each case KRAS^ED^ expression inhibited tumor formation from concomitant activation of the *Braf^CA^* allele relative to the LEX-EGFP-iCL control virus. Figure 3 shows the difference between lung sections of *Braf^CA/+^* mice infected with either LEX-EGFP-iCL or LEX-KRAS^ED^-iCL at 16 weeks post-infection. At 16 weeks post-infection, the lowest number of tumors was observed with KRAS^V12^ (1 tumor in 1 lung out of 2 mice), whereas the KRAS^V12/S35^ and KRAS^V12/E38^ conditions had an average of 8 and 17 tumors per lung, respectively (Table 1). These last two mutants both activate the BRAF-MEK-ERK pathway but not the RalGDS or PI3K/Akt pathways.

**Figure 4:**
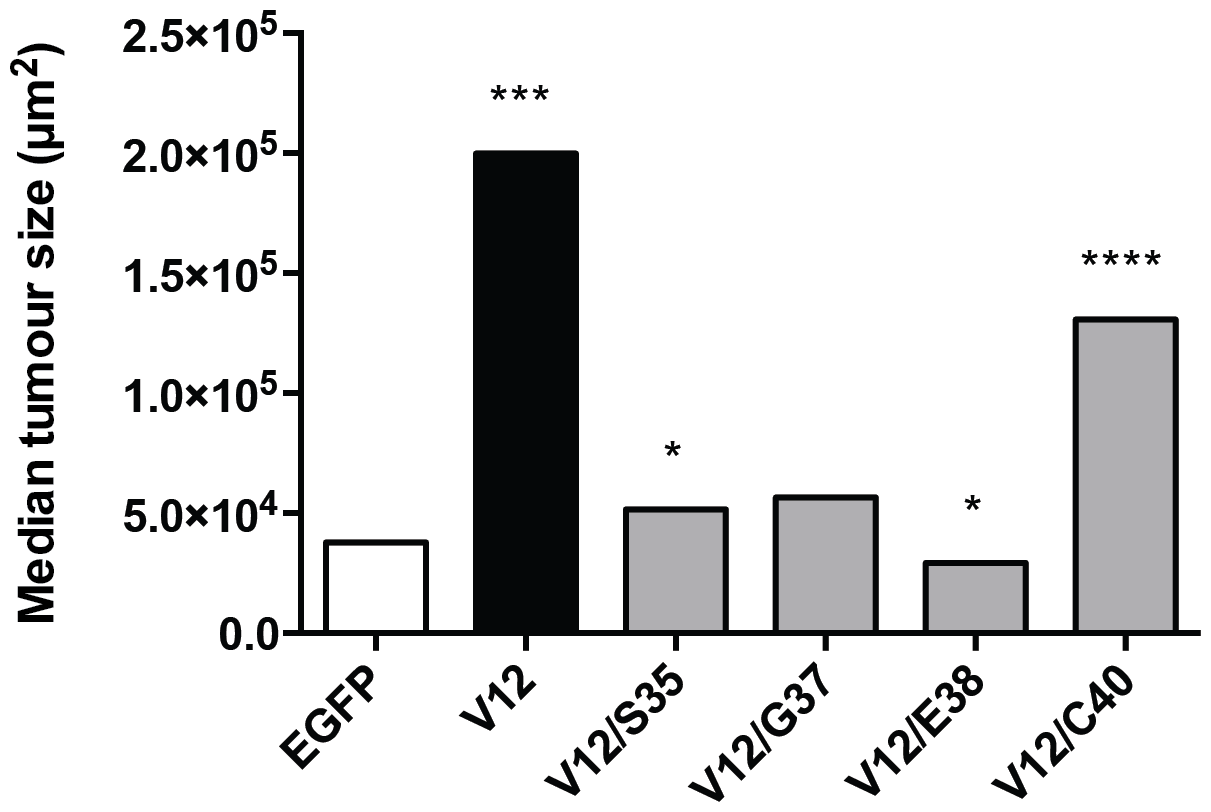
Median tumor size. Tumor size was measured by section area at 16 weeks post-infection along with Cre-mediated activation of the *Braf^CA^* allele. Distributions of tumor size for all the KRAS mutants were compared with the LEX-EGFP-iCL negative control with the Mann-Whitney U test (* p<0.05; *** p<0.001; **** p<0.0001). Note median tumor size was significantly larger in KRAS^V12^ and KRAS^V12/C40^ expressors.

**Figure 5:**
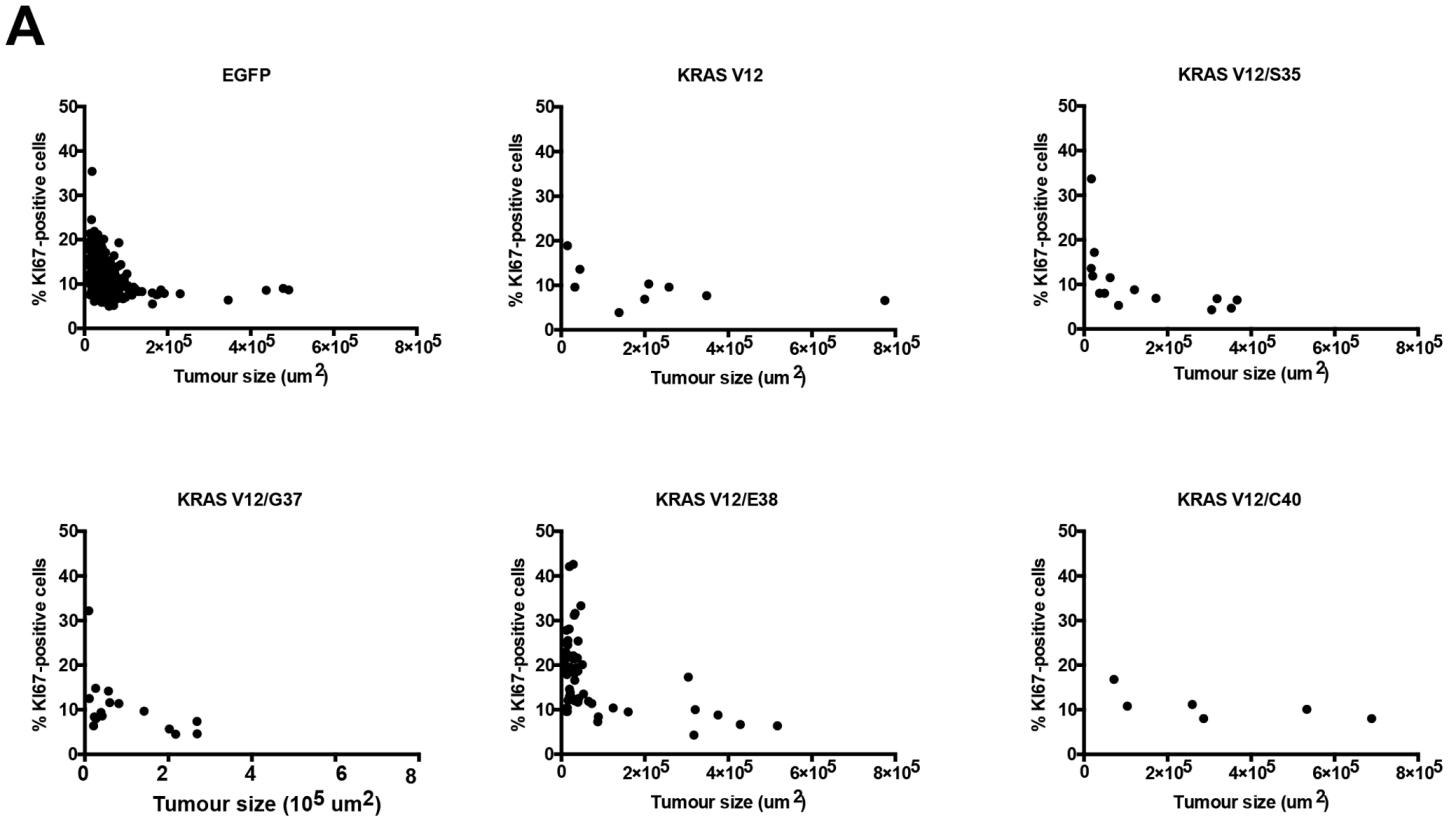
Distribution of size and proliferation of tumors. *Braf^CA/+^* mice were infected with as indicated and tumor size vs. proliferation as measured by Ki67 staining at 16 weeks post-infection. Each dot on the graphs represents a single tumor, for which the surface area and the percentage of KI67-positive cell was determined. The cDNA encoded in each LEX-iCL lentivirus used to infect mice is indicated on each graph.

We sought to determine if other tumor parameters were altered by the expression of different Ras effector mutants. We assessed the median size of the tumors and the distribution of tumor sizes were compared using a two-tailed Mann-Whitney U test between the LEX-EGFP-iCL negative control and the five conditions where KRAS is present (Figure 4). We found that tumors expressing Braf^V600E^ and either KRAS^V12^ or KRAS^V12/C40^ were significantly larger than those expressing Braf^V600E^ alone or Braf^V600E^ and the other KRAS^ED^ mutants. We additionally assessed proliferation rates of these tumors by immunohistochemical staining for Ki67 (supplemental figure S6). The size and proliferative status of each adenoma were plotted and are shown in Figure 5. A general trend was observed where smaller tumors had a high proliferation rate corresponding to the tumor initiation stage, whereas larger tumors had a lower proliferation rate. As expected, the presence of KRAS^V12^ expression resulted in larger tumors relative to the mice receiving LEX-EGFP-iCL (p = 0.0008). The tumors arising from the KRAS^S35^-iCL virus were bigger than in the LEX-EGFP-iCL condition (p = 0.0245), yet the median tumor size for KRAS^S35^ tumors was less than two times that of those obtained with control LEX-EGFP-iCL virus, indicating there wasn't a strong effect on proliferation induced by that mutant. The KRAS^G37^ mutant did not lead to a statistically significant increase in tumor size compared to those seen with EGFP-iCL. Surprisingly, expression of KRAS^E38^, which selectively activates the MAPK pathway, in the *Braf^CA/+^* mice led to smaller tumors than with Braf^V600E^ expression alone (i.e., with LEX-EGFP-iCL infection). This is in contrast with the KRAS^S35^ mutant, which is also activates the MAPK pathway, suggesting an excess of MAPK signalling can lead to either more proliferation or a more abrupt OIS response. The difference in phenotype between the KRAS^S35^ and KRAS^E38^ mutants could be caused by other effectors that they selectively bind to, which could shift the balance either way. Expression of Braf^V600E^ along with KRAS^C40^, which selectively activates the PI3K/AKT pathway, mimicked the expression of KRAS^V12^. Here the median tumors size caused by the LEXKRAS^C40^-iCL virus was more than 3 times larger than with LEX- EGFP -iCL (p<0.0001), although there were fewer tumors (Figure 4).

## Discussion

Here we describe a genetic strategy to concomitantly activate the MAPK pathway through Cre-dependent expression of Braf^V600E^ along with subgroups of KRAS effectors by simultaneously expressing KRAS^V12^ effector domain mutants. Lentiviruses encoding EGFP and Cre recombinase efficiently induced lung adenomas similar to those observed with adenovirus activation of the *Braf^CA^* allele. Co-expression of Braf^V600E^ and activated KRAS significantly reduced tumor formation, with this very low tumor burden precluding our ability to observe progression to the adenocarcinoma stage as would be predicted. It is possible that the elevated expression of KRAS^V12^ coupled with Braf^V600E^ induces senescence at an early stage in tumor development precluding detection.

Sustained elevated Ras-Raf-MEK signalling in human fibroblasts leads to a senescence response with the induction p16INK4A and p21 expression [46]–[49]. There is ample evidence to support the notion of oncogene and stress induced senescence both in mouse models and in human tumors [50]. For example, in a *KRas^V12^*-driven model of lung cancer, the majority of tumors that form are adenomas that express senescent markers along with a low proliferative index. In these mice when adenocarcinomas are observed, they lack these markers [36], [49]. Constitutively active BRAF^V600E^ expression in melanocytes gives rise to nevi (more commonly known as moles), which are benign lesions that typically display hallmarks of senescence. In humans these lesions often remain dormant for decades but can progress to malignant melanoma [51]–[53]. This ability of Raf proteins to induce a growth arrest *in vitro* and *in vivo* is dependent on their expression levels, where lower expression levels can induce proliferation and high levels leads to rapid cell cycle arrest [46], [54] [54]. The correlation between OIS and expression level can additionally be inferred from studying human Spitz nevi, melanocytic lesions, which possess amplification of an activated HRAS allele (most commonly a G12V allele). These lesions have elevated HRAS yet rarely progress to full malignancy [55]. Taken together these data demonstrate that elevated levels of Ras/Raf signalling are linked to senescence induction.

Recent studies have demonstrated an approximately 10-fold higher tumor burden with Braf^V600E^ expression in mouse lung compared to Kras^G12D^, while adenocarcinoma formation was exclusively observed with Kras^G12D^ but not Braf^V600E^ expression [56], [57]. This suggests one or more of the following may be true: that Braf^V600E^ is less efficient at inducing senescence than is Kras^G12D^, that Braf^V600E^ is a more efficient tumor initiator, or that there are fewer cells infected by adenovirus Cre that express Kras when compared to those expressing Braf. Here we have forced KRAS^V12^ expression thus it is likely that the reduced tumor number observed is a consequence of increased engagement of senescence.

### KRAS effector domain mutants

Of the four KRAS effector domain mutants, KRAS^V12/C40^ produced the largest increase in tumor size in *Braf^CA/+^* mice, relative to Braf^V600E^ expression alone. The C40 mutant is the only mutant capable of interacting with the p110 subunit of PI3K [23]. It additionally interacts with Tiam1, RASSF4 and RIN3, as do the S35, G37 and E38 mutants [23], [25]. As the PI3K pathway activation is specific to the C40 mutant, it suggests that this pathway cooperates with constitutive MAPK signalling to increase tumor growth. Of the PI3K genes, only p110α and p110γ are activated by Kras, whereas p110δ is activated by Rras and TC21. The p110 β isoform activation appears to occur independently of the Ras superfamily of GTPases [23]. The PIK3CA gene, coding for the p110α subunit of PI3K, is mutated in 2% of lung adenocarcinomas [7], which suggests that this isoform might be a key interactor of KRAS^V12/C40^ that promoted tumor growth. It will be of interest to formally test the cooperation between activated alleles of PIK3CA and Braf using a newly engineered PIK3CA GEM [58].

Both the S35 and E38 KRAS^ED^ mutants activate MAPK signalling while failing to interact with RalGDS or PI3K yet differences in the phenotypes were observed. Expression of the KRAS^V12/S35^ or KRAS^V12/E38^ mutants permitted the formation of more tumors than the KRAS^V12^, KRAS^V12/G37^ and KRAS^V11/C40^ mutants when coexpressed with Braf^V600E^, yet far fewer than with Braf^V600E^ expression alone. The combined expression of Braf^V600E^ and KRAS^V12/E38^ resulted in smaller tumors than Braf^V600E^ expression alone. In fibroblasts, the levels of activity of Raf-1 determine whether cells enter a proliferative state or a growth state [46]. Moreover the different Raf proteins (Araf, Braf, and Raf-1) display different kinase activities and the levels of induction of a growth arrest correlates with these levels [46]. It is not known if KRAS^V12/S35^ or KRAS^V12/E38^ display different affinities for the three Raf proteins. If so, it can be hypothesized that this differential affinity could results in a premature or delayed senescence response. Another possibility comes from previous protein pull-down experiments, in which the E38 mutant seems to have a greater affinity for RIN and RASSF1 compared to the S35 mutant [23]. RASSF1 is a tumor suppressor that is often silenced in NSCLC [59]. Also, two studies have shown that the S35 mutant, and not E38, is able to bind weakly to RalGDS and RGL, another Ral GEF, although it is still unclear if this is sufficient to cause a physiologically relevant increase in Ral signalling [23], [24]. All of the above suggests that, even though S35 and E38 mutants of KRAS have been used in multiple studies as specific activators of MAPK signalling, they may not have the same functional properties and thus should not be considered as genetic equivalents.

Our data makes it difficult to conclude if RalGDS signalling has a positive effect on NSCLC progression. KRAS^V12/G37^ interacts with more RA domain-containing proteins (including RIN, RIN2, Nore1, RASSF1, RGL, RGL2) than any other effector mutant. Also, as the RASSF family of tumor suppressor proteins all possess an RA domain, it is possible that the G37 mutant, by having a higher affinity for the RA domain than the S35, E38 and C40 mutants, activated a RASSF-dependent tumor suppressive response that counteracted oncogenic Ral signalling [23], [60].

### Oncogene-induced senescence blocking tumor initiation

Our results indicate that lentiviral driven KRAS^V12^ expression concomitant with Braf^V600E^ expression reduces tumor number by over 100-fold compared to Braf^V600E^ expression alone. The most straightforward explanation would be that increased signalling output from KRAS^V12^ lead cells into an oncogene-induced senescence (OIS) response. This notion becomes difficult to reconcile with the observations that decreased tumor formation occurred when each of the KRAS^ED^ mutants were coexpressed along with the *Braf^V600E^*. There is the possibility that the activation of single pathways downstream of KRAS with each effector domain mutant expressed is sufficient to induce OIS. However, this model would fail to explain why the KRAS^V12/S35^ or KRAS^V12/E38^ mutants, which activate the RAF-MEK-ERK MAPK pathway, fail to produce tumors when expressed alone in wild-type mice but do give rise to tumors when expressed concomitantly with an activated *Braf^V600E^* allele. This would be predicted to further increase the MAPK oncogenic signalling output and to elicit a stronger OIS response. None of the KRAS effector domain mutants were capable of causing tumor formation on their own, and KRAS^V12^ expression led to tumor formation in only two wild-type mice out of eight. It is thus improbable that increased downstream oncogenic signalling is the only cause of the abrogation of tumor formation and suggests an additional mechanism to explain this tumor suppression exists.

### Direct activation of a negative regulator by KRAS

The RASSF family of proteins are direct Ras effectors that act as tumor suppressors and can antagonize the antiapoptotic and pro-proliferative effects of Ras. They are frequently found transcriptionally silenced early in the development of various cancers [60]. There are 10 members of the RASSF family proteins in mammals. RASSF1–6 possess a C-terminal Ras association (RA) domain, whereas RASSF7–10 possess an N-terminal RA domain [60], [61]. RASSF1–6 proteins also share a Sav-RASSF-Hpo (SARAH) domain, which interacts with the proapoptotic MST1/2 kinases [60]. There are several lines of evidence that points to a role of RASSF proteins mediating negative signal downstream of Ras.

*Drosophila* possesses a single RASSF ortholog, dRASSF, which shares a C-terminal RA domain and a SARAH domain like its mammalian RASSF1–6 counterparts. Through the SARAH domain *Drosophila* dRASSF physically interacts with Hippo (Hpo), which is homologous to mammalian MST1/2. Hpo is a central protein kinase of the Salvador/Warts/Hippo pathway controlling organ size in animals through regulation of cell proliferation and apoptosis [62]. Hpo and MST1/2 kinases are negative regulators of the *Drosophila* Yorkie or mammalian YAP/TAZ proteins respectively, which function to activate transcription of anti-apoptotic and pro-proliferative genes. *Hpo* loss in *Drosophila* results in tissue overgrowth [62], which phenocopies elevated Yorkie expression [62], [63]. Similarly, mouse *Mst1/2* deficiency in the liver results in the loss of inhibition of Yap1, massive liver overgrowth and hepatocellular carcinoma formation [64]. Although biochemical evidence in *Drosophila* points to an inactivation of the Hpo kinase by dRASSF, concomitant loss of *Hpo* and *dRASSF* enhances the tissue overgrowth phenotype observed with *Hpo* loss alone, suggesting a tumor suppressor function for dRASSF that is independent of its interaction with Hpo [65]. Moreover loss of *Ras1* in *Drosophila* reduces cell growth and increases apoptosis [65], [66], while ommatidia-specific loss of function *dRASSF* alleles rescue the growth defects observed with mutant *Ras1* alone by increasing proliferation and decreasing apoptosis [65]. Together, this suggests that dRASSF is a genetic antagonist of Ras signalling in *Drosophila*.

In humans, RASSF1, RASSF3, RASSF4 and RASSF5 each are implicated in mediating a negative signal to repress proliferation and/or survival. Of these, *RASSF1A* has received the most attention. Its promoter is found methylated and inactivated in a variety of cancers and in particular in 33% of NSCLC. This is associated with poor overall relapse-free survival. Moreover, *RASSF1A* has the properties of a tumor suppressor gene [59], [67]. RASSF1A-specific knockout mice, while fully viable and fertile, display increased spontaneous- and carcinogen- induced tumor susceptibilities, particularly in the lung [68], [69]. Moreover, Raf1 inhibits MST2 activity thus preventing apoptosis [70]. Expression of RASSF1A can disrupt MST2-Raf1 association thereby relieving this Mst2 inhibition and inducing apoptosis [71]. Additionally *RASSF4* is located in a region with frequent LOHs observed in prostate cancer [72] and it is mutated in lung and breast cancers [73]. When tethered to the plasma membrane via a CAAX membrane localization sequence, RASSF4 induces apoptosis [73]. RASSF3 is a Ras-dependent proapoptotic protein [74] that is frequently found transcriptionally silenced in many cancers including in 24% of NSCLC [67], [75], [76] and has been identified in a screen for genes that suppress MMTV-Neu-driven breast cancer [77]. Finally, RASSF3 can bind MST1/2 via its SARAH domain [74], which is also used by other RASSF proteins (RASSF1A, RASSF2, RASSF5) to induce apoptosis [78], [79]. Taken together these data suggest that at least four RASSF family members can function as negative regulators of tumorigenesis.

While the methylation and mutational status the other RASSF family members is as yet unknown it is interesting to note that *RASSF7*, *RASSF8* and *RASSF10* genes are in close proximity with the *HRAS*, *KRAS* and *RRAS* loci respectively, suggesting a potential common evolution with Ras GTPases [61]. It should be noted that while binding to KRAS^ED^ mutants has not been evaluated for all the RASSSF members, RASSF4 binds all the KRAS^ED^ Mutants (S35, G37, E38, C40) [23]. If other RASSF proteins display similar binding this could provide an explanation for the decreased tumor reduction phenotype observed in each condition where KRAS^V12^ alleles is present. Taken with the above data, one can speculate that the decrease tumor formation we see with each KRAS^ED^ mutant could be due to recruitment of one or more RASSF-members to the activated KRAS alleles. Perhaps this was a consequence of the levels of KRAS^ED^ mutant expression obtained from lentiviral expression.

Further studies can be done to test this notion. A cleverly conceived transgenic mouse (called *RasE* Multi-Hit) has been recently developed to study which pathways may cooperate to induce HRAS-driven cancer [80]. This allele codes for three effector domain mutants (HRAS^V12/S35^, HRAS^V12/G37^, HRAS^V12/C40^) each of which may be expressed at physiological levels upon Cre mediated recombination in a stochastic manner [80]. This approach allows for the expression of one, two or three HRAS^V12-ED^ mutants. The majority of lung tumors formed after Cre activation expressed all three alleles thus simultaneously activating the MAPK, PI3K and RalGDS pathways. Interestingly, no tumors were found coexpressing the HRas^V12/G37^ and HRas^V12/C40^ in the absence of HRas^V12/S35^, even though individually the G37 and C40 alleles were found in 0.6% and 6% of the tumors formed respectively. This is another indication supporting the hypothesis that the sum of the signalling pathways activated by Ras leads to a direct increase in both pro-proliferative and tumor suppressive functions.

## Materials and Methods

### Ethics Statement

Administration of lentiviral vectors and subsequent euthanasia was performed under 2,2,2 Tribromoethanol anaesthesia. All efforts were made to minimize suffering throughout the course of these studies. All mouse experiments were carried out in strict accordance with the recommendations in the Canadian Council on Animal Care (CCAC) “*Guide to the Care and Use of Experimental Animals*” and under the conditions and procedures approved by the Animal Care Committee of McGill University (AUP number: 5819).

### Cloning and mutagenesis

Human KRAS 4B cDNA was PCR amplified from a cDNA provided by Pablo Rodriguez-Viciana (University College of London) using forward primer 5′-CACCGGATCCACCATGACTGAATATAAACTTGTGG and reverse primer 5′-CTCGAGAGATCTCAATTGTTACATAATTACACACTTTGTCTTTG with the Phusion polymerase (Thermo scientific), as per the manufacturer's instructions. The PCR product was cloned into pENTR/D-TOPO (Invitrogen) following manufactures instructions.

Mutagenesis of the KRAS cDNA was performed using overlapping forward and reverse primers that included the desired mutation, using the Phusion DNA polymerase (Thermo scientific). The following primers pairs were used for mutatgenesis to create the G12V (GV0004 fwd, GGTAGTTGGAGCCGTGGGCGTAGGCAAG; GV0005 rev, CTTGCCTACGCCCACGGCTCCAACTACC), T35S (GV0006 fwd, GGACGAATATGATCCGTCGATAGAGGATTCC; GV0007 rev GGAATCCTCTATCGACGGATCATATTCGTCC), E37G (GV0008 fwd, CGAATATGATCCTACCATAGGGGATTCCTACAG; GV0009 rev, CTGTAGGAATCCCCTATGGTAGGATCATATTCG), D38E (GV0010 fwd, GATCCAACAATAGAAGAGTCCTACAGGAAG; GV0011 rev, CTTCCTGTAGGACTCTTCTATTGTTGGATC) and Y40C (GV0012 fwd, CATAGAGGATTCCTGCAGGAAGCAAGTAG; GV0013 rev, CTACTTGCTTCCTGCAGGAATCCTCTATG) mutations. Nucleotides that differ from that in the wild-type sequence are underlined. Following a 30 second 98°C denaturation step, 16 cycles of PCR were performed (30 sec at 98°C, 1 min at 55°C, 2 min at 72°C), followed by a 10 min incubation at 72°C. 20 units of DpnI enzyme was added to the cooled PCR product. Following a 1.5 hr 37°C incubation, the mixture was heated to 80°C for 20 min and a portion was transformed into competent bacteria. All cloned PCR-amplified regions were sequenced in their entirety,

#### Lentiviral vector

To facilitate cloning a Gateway compatible Lentiviral vector, gLEX-iCL, was created. gLEX-iCL contains the Gateway selection cassette transcriptionally upstream of an internal ribosome entry sequence followed by CreLuc fusion protein. The Not1/HpaI fragment containing ires-puro was replaced with ires-CruLuc, from pENTR-ires-CruLuc [41]. CreLuc was created to express both Cre recombinase and firefly luciferase separated by a *Thosea asigna* virus-derived 2A peptide (T2A ‘translational slip’) sequence [39], [40] for polycistronic expression. EGFP, KRAS^G12^ and the KRAS effector domain mutants were cloned into pENTRd-TOPO and Gateway LR reactions were used to produce lentiviral expression vectors containing these cDNAs.

### Cell Culture

#### Cell Culture

HEK 293T and derivatives were cultured in DMEM (Wisent) containing 10% FBS, 1% penicillin/streptomycin (Wisent) and 1% v/v 1M HEPES solution at 37°C with 5% CO2. Cells were trypsinized and split 1∶10 into fresh plates at regular intervals to prevent them from reaching confluence. L9.2 cells are a Cre reporter cell line made by transfecting CALNL-dsRed (described in [81]) into 293 cells and selecting for stable integration of this plasmid via G418 selection. G418-resistant cells were plated at clonal density and individual clones were isolated and tested for Cre-dependent dsRed expression. The cell line L9.2 was derived by cloning the line two successive times (not shown). Cells were infected with equal amounts of virus with 4 µg/ml of polybrene and imaged 72 hours post-infection to detect dsRed expression resulting from Cre recombination.

### Lentivirus Production and Quantification

#### Lentivirus Production and Purification

Lentivirus was produced in HEK 293T cells by co-transfection using a Polyethyleneimine (P.E.I.) solution at a 2.65∶1 ratio (P.E.I. mass∶DNA mass). Specifically ten 175 cm^2^ tissue culture-treated vented flasks were seeded with 8×10^6^ HEK 293T cells and were transfected the following day with psPAX2 packaging plasmid (11.7 µg), pMD2.G envelope plasmid containing VSV-G (6.3 µg), the recombinant lentiviral plasmid (18 µg) and 95.9 ug P.E.I. per plate as described [82]. 16 hours post-transfection the media was changed to 20 ml DMEM containing 10% FBS, 1% Penn/Strep and 50 mM HEPES pH 7.3. 24 hr later the supernatant was collected, filtered through 0.22 µm bottle-top filter (Millipore) and concentrated by ultracentrifugation through a 20% sucrose cushion for 2 hr at 4°C using a Beckman Coulter SW32 Ti rotor at 29,500 RPM (82,000×g) [83]. Lentiviral pellets were resuspended in 600 ul 1×PBS at 4°C for two hours with occasional gentle vortexing. Concentrated virus was aliquoted and immediately stored at −80°C.

#### Lentivirus titration - Flow Cytometry

Flow cytometry was used to titre the EGFP-iCL virus and the infectious titre obtained was correlated with the RT-qPCR data obtained from the same viral preparations. 6-well dishes were seeded with 1×10^5^ 293T cells per well. The following day, cells were infected with 10 or 100 µl of 1∶10 and 1∶100 dilutions of the virus in individual wells in the presence 4 µg/ml of polybrene. The following day, the medium was changed and the proportion of EGFP-positive cells was determined by standard flow cytometry analysis 72 hours post-infection using a Becton Dickinson FACScan with the 530/30 filter. The infectious titre of each virus dilution could be calculated as (titre = %EGFP+ cells×number of infected cells×dilution factor)/(volume of virus tested).

#### Lentivirus titration - RT-qPCR

RNA was extracted from 1∶10 and 1∶100 dilutions of viruses using an RNeasy kit (QIAGEN) according to the manufacturer's instructions. Potential DNA carryover was removed with a 15 minute treatment with DNAseI (Invitrogen). cDNA was generated using the GoScript Reverse Transcription kit (Promega) according to the manufacturer's instructions, except for the use of a specific oligonucleotide (5′-GCAGAATCCAGGTGGCAACA) to prime cDNA synthesis to replace poly d(T) or random primers.

cDNA concentration was determined through quantitative PCR (qPCR). A standard curve was produced each time using 10 fold dilutions spanning 10 ng/ul to 10 fg/ul of pLEX-EGFP-iCL plasmid DNA as an internal control. The primers and probe used were 5′-CCTTTCCGGGACTTTCGCTTT (GV0022 fwd), 5′-GCAGAATCCAGGTGGCAACA (GV0022 rev) and 5′/6-FAM/ACTCATCGC/ZEN/CGCCTGCCTTGCC/IABkFQ/-3′ (probe), as described previously [42].

### Lentiviral infections of mouse lungs

#### Strains and genotyping

The *BrafCA* mouse strain, described previously [35], was backcrossed 7 times in a C57BL/6 background. Matings were set up as to obtain both *BrafCA/+* and wild-type mice in a 1∶1 ratio for the different experiments. Genomic DNA for PCR was extracted as described [84] and the *Braf^CA^* allele was identified by PCR as described [35].

#### Anaesthetic preparation

A 1 g/ml solution of Avertin (2,2,2-tribromoethanol, Sigma) was made in 2-methyl-2-butanol. This solution was then diluted to 25 mg/ml with a sterile solution of 1 mM Tris-HCl pH 7.4, 250 uM EDTA and 137 mM NaCl and subsequently 0.22 µm filtered prior to use. While use of isoflorane would be preferred we were unable to obtain sufficient anaesthesia to routinely administer lentiviruses.

#### Mouse lung infections

The mice used for the lung infections were all female FVB/NJ mice. Mice were anesthetized by intraperitoneal injection of Avertin at a dose corresponding to 0.3 mg of Avertin per gram of mouse weight. 25 µl of 40 mM sodium caprate (Sigma) in 1×PBS was administered either by intranasal instillation or by using an I.V. catheter (Becton Dickinson, cat. #381223), followed by 62.5 µl of lentivirus (2×10^7^–10^8^ IU [intranasal]; 10^8^ IU [tracheal intubation]) ten minutes later. During the procedure and up until recovery, the mice were kept on a 37°C pad to prevent hypothermia. Mice were euthanized at 18 to 19 weeks post-infection to harvest the lungs for analysis.

### Histology analysis

#### Tissue processing

Lungs were perfused with 1×PBS, fixed at 4°C in zinc formalin (Sigma) for 24 hours and subsequently embedded in paraffin. The paraffin blocks were cut in 5 µm sections by step-sectioning at 200 µm intervals.

#### Histopathological analysis

Lung sections were stained using a standard haematoxylin & eosin (H&E) staining protocol. For the KI67 immunohistochemistry, antigen retrieval step was performed by pressure-cooking the slides in 10 mM sodium citrate, pH 6.0 for 10 minutes. Slides were blocked with a 2% BSA/1×PBS, incubated overnight with the primary antibody (Abcam #ab15580) at 1∶1000 dilution and then washed once in 1×PBS and twice in PBST (1×PBS with 0.05% Tween-20). Endogenous peroxidises were blocked with a 20 minute incubation in 3% H2O2. Slides were washed once with distilled H2O, twice in 1×PBS for and then blocked with 2% BSA/1×PBS for 1 hr. Sections were incubated for 45 minutes at room temperature with a biotin-conjugated donkey anti-rabbit secondary antibody (Jackson ImmunoResearch #711-065-152) diluted to 1∶500 in the 2% BSA/1×PBS solution. The slides were then washed in distilled H2O and then twice with TBST prior to the application of the biotin-streptavidin ABC complex complex (Vectastain #PK-4000) according to the manufacturer's instructions. The slides were washed three times in PBS and the staining was revealed using DAB (Invitrogen #750118). The slides were thereafter counterstained with haematoxylin as described [35].

Slides stained with H&E and Ki67 were scanned using an Aperio Scanscope AT. Individual slides were analyzed using the Aperio ImageScope software, in which each tumor was circumscribed to obtain the section area (µm^2^) and the percentage of Ki67-positive cells was obtained using the IHC Nuclear Algorithm.

## Supporting Information

Figure S1 **Infection of 293T L9.2 Cre reporter cells that express dsRed upon Cre expression.** In each condition, 2.5×10^5^ cells were infected with 6.9×10^6^ IU the same viruses that were used to infect mice.
(TIF)

Figure S2 **Focus formation in 3T3 cells stably expressing the different KRAS effector domain mutants.** 1×10^4^ cells of each stable cell line were seeded with 2.5×10^6^ of the parental 3T3 C5 cell line and left at confluency for 14 days. In the KRAS^V12^ condition, 1×10^3^ cells were seeded instead of 1×104 cells to be able to count single foci and the number was normalized thereafter. Error bars: standard error of the means (SEM).
(TIF)

Figure S3 **Colony formation in soft agar of 3T3 cell lines expressing the KRAS effector domain mutants.** In each well, 1×10^4^ cells were seeded in DMEM with 0.35% low melting point agarose. After 14 days, the number of colonies were stained with MTT and counted. Error bars: SEM.
(TIF)

Figure S4 **Determining titre of concentrate lentivirus.** **A**) Schematic representation of pLEX-eGFP-ires Cre(2a)Luc (pLEX-eGFP-iCL), a gLEXiCL-derived that expresses eGFP transcriptionally upstream of the Cre(2a)Luc fusion. Indicated is the location of the PCR primers used to quantify lentiviral RNA-derived molecules. **B**) Seven independent LEX-eGFP-iCL lentiviral preparations were generated and were concentrated, with a small aliquot of preps 1–5 being frozen directly. These viral preps were split, diluted and used to infect 293T cells or to isolate RNA for RT PCR analysis. The graph represents correlation between infectious units, as judged by GFP positivity with FACS analysis, and RNA molecules, by RT PCR. Asterisks indicate the values obtained for undiluted virus and are color-coded with the corresponding prep the figure legend. Preps 6 and 7 were not tested as undiluted viruses. The dotted line indicates the line of best fit the correlation coefficient of r = 0.93.
(TIF)

Figure S5 **Lentiviral and Adenoviral activation of Braf^CA^ produce similar tumors.** **A**) Braf^+/+^ or **B**) Braf^CA/+^ mice were infected with 10^8^ IU of pLEX-EGFP-iCL lentivirus and lung tissue was obtained 16 weeks postinfection. The tissue of was analysed by immunoflourescence for Clara Cell Antigen (CCA, in red), which marks Clara cells and Surfactant Protein C (SPC, in green), which marks type II pneumocytes. Nuclei are stained blue with DAPI. **C**) Braf^CA/+^ mice were infected with 5×10^6^ PFU of Adenoviral Cre and analysed as in B). Note tumors (marked by arrowheads) initiated with either adenovirus or lentivirus stain negative for CCA and positive for SPC.
(TIF)

Figure S6 **Representative staining for Ki67 determination.** Representative images of **A**) H&E and **B**) Ki67 stained lung tissues at low (upper) and high (lower) magnification. Aperio software was used to quantify the percentage of Ki67 positive nuclei. Analysis was focused on individual tumours, which were manually were circled (6 tumors are shown circled in green as an example).
(TIF)

Methods S1 (DOCX)

Table S1 (DOCX)

## References

[1] Ferlay J, Shin HR, Bray F, Forman D, Mathers C, et al.. (2008) GLOBOCAN 2008 v2.0, Cancer Incidence and Mortality Worldwide: IARC CancerBase No. 10. Lyon, France: International Agency for Research on Cancer; 2010.

[2] Sun S, Schiller JH, Gazdar AF (2007) Lung cancer in never smokers–a different disease. Nat Rev Cancer 7: 778–790. 17882278 10.1038/nrc2190

[3] Goldstraw P, Crowley J, Chansky K, Giroux DJ, Groome PA, et al (2007) The IASLC Lung Cancer Staging Project: proposals for the revision of the TNM stage groupings in the forthcoming (seventh) edition of the TNM Classification of malignant tumours. J Thorac Oncol 2: 706–714. 17762336 10.1097/JTO.0b013e31812f3c1a

[4] Travis WD, Brambilla E, Noguchi M, Nicholson AG, Geisinger KR, et al (2011) International association for the study of lung cancer/american thoracic society/european respiratory society international multidisciplinary classification of lung adenocarcinoma. J Thorac Oncol 6: 244–285. 21252716 10.1097/JTO.0b013e318206a221 PMC4513953

[5] Little AG, Rusch VW, Bonner JA, Gaspar LE, Green MR, et al (2005) Patterns of surgical care of lung cancer patients. Ann Thorac Surg 80: 2051–2056 discussion 2056. 16305843 10.1016/j.athoracsur.2005.06.071

[6] Wong DW, Leung EL, So KK, Tam IY, Sihoe AD, et al (2009) The EML4-ALK fusion gene is involved in various histologic types of lung cancers from nonsmokers with wild-type EGFR and KRAS. Cancer 115: 1723–1733. 19170230 10.1002/cncr.24181

[7] Bamford S, Dawson E, Forbes S, Clements J, Pettett R, et al (2004) The COSMIC (Catalogue of Somatic Mutations in Cancer) database and website. Br J Cancer 2004/06/10 ed 355–358. 15188009 10.1038/sj.bjc.6601894 PMC2409828

[8] Seo JS, Ju YS, Lee WC, Shin JY, Lee JK, et al (2012) The transcriptional landscape and mutational profile of lung adenocarcinoma. Genome Res 10.1101/gr.145144.112 PMC3483540 22975805

[9] Pao W, Miller V, Zakowski M, Doherty J, Politi K, et al (2004) EGF receptor gene mutations are common in lung cancers from “never smokers” and are associated with sensitivity of tumors to gefitinib and erlotinib. Proc Natl Acad Sci U S A 101: 13306–13311. 15329413 10.1073/pnas.0405220101 PMC516528

[10] Paez JG, Janne PA, Lee JC, Tracy S, Greulich H, et al (2004) EGFR mutations in lung cancer: correlation with clinical response to gefitinib therapy. Science 304: 1497–1500. 15118125 10.1126/science.1099314

[11] Rodig SJ, Shapiro GI (2010) Crizotinib, a small-molecule dual inhibitor of the c-Met and ALK receptor tyrosine kinases. Curr Opin Investig Drugs 11: 1477–1490. 21154129

[12] Soda M, Choi YL, Enomoto M, Takada S, Yamashita Y, et al (2007) Identification of the transforming EML4-ALK fusion gene in non-small-cell lung cancer. Nature 448: 561–566. 17625570 10.1038/nature05945

[13] Gandhi L, Janne PA (2012) Crizotinib for ALK-rearranged non-small cell lung cancer: a new targeted therapy for a new target. Clin Cancer Res 18: 3737–3742. 22547770 10.1158/1078-0432.CCR-11-2393

[14] Eberhard DA, Johnson BE, Amler LC, Goddard AD, Heldens SL, et al (2005) Mutations in the epidermal growth factor receptor and in KRAS are predictive and prognostic indicators in patients with non-small-cell lung cancer treated with chemotherapy alone and in combination with erlotinib. J Clin Oncol 23: 5900–5909. 16043828 10.1200/JCO.2005.02.857

[15] Gaughan EM, Costa DB (2011) Genotype-driven therapies for non-small cell lung cancer: focus on EGFR, KRAS and ALK gene abnormalities. Ther Adv Med Oncol 3: 113–125. 21904575 10.1177/1758834010397569 PMC3150063

[16] Riely GJ, Marks J, Pao W (2009) KRAS mutations in non-small cell lung cancer. Proc Am Thorac Soc 6: 201–205. 19349489 10.1513/pats.200809-107LC

[17] Laurent-Puig P, Lievre A, Blons H (2009) Mutations and response to epidermal growth factor receptor inhibitors. Clin Cancer Res 15: 1133–1139. 19228718 10.1158/1078-0432.CCR-08-0905

[18] Pylayeva-Gupta Y, Grabocka E, Bar-Sagi D (2011) RAS oncogenes: weaving a tumorigenic web. Nat Rev Cancer 11: 761–774. 21993244 10.1038/nrc3106 PMC3632399

[19] Karnoub AE, Weinberg RA (2008) Ras oncogenes: split personalities. Nat Rev Mol Cell Biol 9: 517–531. 18568040 10.1038/nrm2438 PMC3915522

[20] Scheffzek K, Ahmadian MR, Kabsch W, Wiesmuller L, Lautwein A, et al (1997) The Ras-RasGAP complex: structural basis for GTPase activation and its loss in oncogenic Ras mutants. Science 277: 333–338. 9219684 10.1126/science.277.5324.333

[21] Buhrman G, Holzapfel G, Fetics S, Mattos C (2010) Allosteric modulation of Ras positions Q61 for a direct role in catalysis. Proc Natl Acad Sci U S A 107: 4931–4936. 20194776 10.1073/pnas.0912226107 PMC2841912

[22] Pacold ME, Suire S, Perisic O, Lara-Gonzalez S, Davis CT, et al (2000) Crystal structure and functional analysis of Ras binding to its effector phosphoinositide 3-kinase gamma. Cell 103: 931–943. 11136978 10.1016/s0092-8674(00)00196-3

[23] Rodriguez-Viciana P, Sabatier C, McCormick F (2004) Signaling specificity by Ras family GTPases is determined by the full spectrum of effectors they regulate. Mol Cell Biol 24: 4943–4954. 15143186 10.1128/MCB.24.11.4943-4954.2004 PMC416418

[24] Rodriguez-Viciana P, Warne PH, Khwaja A, Marte BM, Pappin D, et al (1997) Role of phosphoinositide 3-OH kinase in cell transformation and control of the actin cytoskeleton by Ras. Cell 89: 457–467. 9150145 10.1016/s0092-8674(00)80226-3

[25] Lambert JM, Lambert QT, Reuther GW, Malliri A, Siderovski DP, et al (2002) Tiam1 mediates Ras activation of Rac by a PI(3)K-independent mechanism. Nat Cell Biol 4: 621–625. 12134164 10.1038/ncb833

[26] Ponting CP, Benjamin DR (1996) A novel family of Ras-binding domains. Trends Biochem Sci 21: 422–425. 8987396 10.1016/s0968-0004(96)30038-8

[27] Kuriyama M, Harada N, Kuroda S, Yamamoto T, Nakafuku M, et al (1996) Identification of AF-6 and canoe as putative targets for Ras. J Biol Chem 271: 607–610. 8557659 10.1074/jbc.271.2.607

[28] White MA, Nicolette C, Minden A, Polverino A, Van Aelst L, et al (1995) Multiple Ras functions can contribute to mammalian cell transformation. Cell 80: 533–541. 7867061 10.1016/0092-8674(95)90507-3

[29] Joneson T, White MA, Wigler MH, Bar-Sagi D (1996) Stimulation of membrane ruffling and MAP kinase activation by distinct effectors of RAS. Science 271: 810–812. 8628998 10.1126/science.271.5250.810

[30] Khosravi-Far R, White MA, Westwick JK, Solski PA, Chrzanowska-Wodnicka M, et al (1996) Oncogenic Ras activation of Raf/mitogen-activated protein kinase-independent pathways is sufficient to cause tumorigenic transformation. Mol Cell Biol 16: 3923–3933. 8668210 10.1128/mcb.16.7.3923 PMC231389

[31] Kwon MC, Berns A (2013) Mouse models for lung cancer. Mol Oncol 7: 165–177. 23481268 10.1016/j.molonc.2013.02.010 PMC5528410

[32] Farago AF, Snyder EL, Jacks T (2012) SnapShot: Lung cancer models. Cell 149: 246–246 e241. 22464334 10.1016/j.cell.2012.03.015

[33] Jackson EL, Willis N, Mercer K, Bronson RT, Crowley D, et al (2001) Analysis of lung tumor initiation and progression using conditional expression of oncogenic K-ras. Genes Dev 15: 3243–3248. 11751630 10.1101/gad.943001 PMC312845

[34] Guerra C, Mijimolle N, Dhawahir A, Dubus P, Barradas M, et al (2003) Tumor induction by an endogenous K-ras oncogene is highly dependent on cellular context. Cancer Cell 4: 111–120. 12957286 10.1016/s1535-6108(03)00191-0

[35] Dankort D, Filenova E, Collado M, Serrano M, Jones K, et al (2007) A new mouse model to explore the initiation, progression, and therapy of BRAFV600E-induced lung tumors. Genes Dev 21: 379–384. 17299132 10.1101/gad.1516407 PMC1804325

[36] Collado M, Gil J, Efeyan A, Guerra C, Schuhmacher AJ, et al (2005) Tumour biology: senescence in premalignant tumours. Nature 436: 642. 16079833 10.1038/436642a

[37] Ji H, Wang Z, Perera SA, Li D, Liang MC, et al (2007) Mutations in BRAF and KRAS converge on activation of the mitogen-activated protein kinase pathway in lung cancer mouse models. Cancer Res 67: 4933–4939. 17510423 10.1158/0008-5472.CAN-06-4592

[38] Fisher GH, Wellen SL, Klimstra D, Lenczowski JM, Tichelaar JW, et al (2001) Induction and apoptotic regression of lung adenocarcinomas by regulation of a K-Ras transgene in the presence and absence of tumor suppressor genes. Genes Dev 15: 3249–3262. 11751631 10.1101/gad.947701 PMC312852

[39] de Felipe P, Luke GA, Hughes LE, Gani D, Halpin C, et al (2006) E unum pluribus: multiple proteins from a self-processing polyprotein. Trends Biotechnol 24: 68–75. 16380176 10.1016/j.tibtech.2005.12.006

[40] Trichas G, Begbie J, Srinivas S (2008) Use of the viral 2A peptide for bicistronic expression in transgenic mice. BMC Biol 6: 40. 18793381 10.1186/1741-7007-6-40 PMC2553761

[41] Geiling B, Vandal G, Posner AR, de Bruyns A, Dutchak KL, et al (2013) A modular lentiviral and retroviral construction system to rapidly generate vectors for gene expression and gene knockdown in vitro and in vivo. PLoS One 8: e76279. 24146852 10.1371/journal.pone.0076279 PMC3795761

[42] Kutner RH, Zhang XY, Reiser J (2009) Production, concentration and titration of pseudotyped HIV-1-based lentiviral vectors. Nat Protoc 4: 495–505. 19300443 10.1038/nprot.2009.22

[43] Lizee G, Aerts JL, Gonzales MI, Chinnasamy N, Morgan RA, et al (2003) Real-time quantitative reverse transcriptase-polymerase chain reaction as a method for determining lentiviral vector titers and measuring transgene expression. Hum Gene Ther 14: 497–507. 12718761 10.1089/104303403764539387

[44] Johnson LG, Vanhook MK, Coyne CB, Haykal-Coates N, Gavett SH (2003) Safety and efficiency of modulating paracellular permeability to enhance airway epithelial gene transfer in vivo. Hum Gene Ther 14: 729–747. 12804137 10.1089/104303403765255138

[45] Jackson EL, Olive KP, Tuveson DA, Bronson R, Crowley D, et al (2005) The differential effects of mutant p53 alleles on advanced murine lung cancer. Cancer Res 65: 10280–10288. 16288016 10.1158/0008-5472.CAN-05-2193

[46] Woods D, Parry D, Cherwinski H, Bosch E, Lees E, et al (1997) Raf-induced proliferation or cell cycle arrest is determined by the level of Raf activity with arrest mediated by p21Cip1. Mol Cell Biol 17: 5598–5611. 9271435 10.1128/mcb.17.9.5598 PMC232408

[47] Zhu J, Woods D, McMahon M, Bishop JM (1998) Senescence of human fibroblasts induced by oncogenic Raf. Genes Dev 12: 2997–3007. 9765202 10.1101/gad.12.19.2997 PMC317194

[48] Lin AW, Barradas M, Stone JC, van Aelst L, Serrano M, et al (1998) Premature senescence involving p53 and p16 is activated in response to constitutive MEK/MAPK mitogenic signaling. Genes Dev 12: 3008–3019. 9765203 10.1101/gad.12.19.3008 PMC317198

[49] Serrano M, Lin AW, McCurrach ME, Beach D, Lowe SW (1997) Oncogenic ras provokes premature cell senescence associated with accumulation of p53 and p16INK4a. Cell 88: 593–602. 9054499 10.1016/s0092-8674(00)81902-9

[50] Kuilman T, Michaloglou C, Mooi WJ, Peeper DS (2010) The essence of senescence. Genes Dev 24: 2463–2479. 21078816 10.1101/gad.1971610 PMC2975923

[51] Michaloglou C, Vredeveld LC, Soengas MS, Denoyelle C, Kuilman T, et al (2005) BRAFE600-associated senescence-like cell cycle arrest of human naevi. Nature 436: 720–724. 16079850 10.1038/nature03890

[52] Dankort D, Curley DP, Cartlidge RA, Nelson B, Karnezis AN, et al (2009) Braf(V600E) cooperates with Pten loss to induce metastatic melanoma. Nat Genet 41: 544–552. 19282848 10.1038/ng.356 PMC2705918

[53] Dhomen N, Reis-Filho JS, da Rocha Dias S, Hayward R, Savage K, et al (2009) Oncogenic Braf induces melanocyte senescence and melanoma in mice. Cancer Cell 15: 294–303. 19345328 10.1016/j.ccr.2009.02.022

[54] Sarkisian CJ, Keister BA, Stairs DB, Boxer RB, Moody SE, et al (2007) Dose-dependent oncogene-induced senescence in vivo and its evasion during mammary tumorigenesis. Nat Cell Biol 9: 493–505. 17450133 10.1038/ncb1567

[55] Maldonado JL, Timmerman L, Fridlyand J, Bastian BC (2004) Mechanisms of cell-cycle arrest in Spitz nevi with constitutive activation of the MAP-kinase pathway. Am J Pathol 164: 1783–1787. 15111324 10.1016/S0002-9440(10)63736-4 PMC1615645

[56] Andreadi C, Cheung LK, Giblett S, Patel B, Jin H, et al (2012) The intermediate-activity (L597V)BRAF mutant acts as an epistatic modifier of oncogenic RAS by enhancing signaling through the RAF/MEK/ERK pathway. Genes Dev 26: 1945–1958. 22892241 10.1101/gad.193458.112 PMC3435497

[57] Trejo CL, Juan J, Vicent S, Sweet-Cordero A, McMahon M (2012) MEK1/2 inhibition elicits regression of autochthonous lung tumors induced by KRASG12D or BRAFV600E. Cancer Res 72: 3048–3059. 22511580 10.1158/0008-5472.CAN-11-3649 PMC3393094

[58] Kinross KM, Montgomery KG, Kleinschmidt M, Waring P, Ivetac I, et al (2012) An activating Pik3ca mutation coupled with Pten loss is sufficient to initiate ovarian tumorigenesis in mice. J Clin Invest 122: 553–557. 22214849 10.1172/JCI59309 PMC3266789

[59] Wang J, Wang B, Chen X, Bi J (2011) The prognostic value of RASSF1A promoter hypermethylation in non-small cell lung carcinoma: a systematic review and meta-analysis. Carcinogenesis 32: 411–416. 21156971 10.1093/carcin/bgq266

[60] Richter AM, Pfeifer GP, Dammann RH (2009) The RASSF proteins in cancer; from epigenetic silencing to functional characterization. Biochim Biophys Acta 1796: 114–128. 19344752 10.1016/j.bbcan.2009.03.004

[61] Underhill-Day N, Hill V, Latif F (2011) N-terminal RASSF family: RASSF7-RASSF10. Epigenetics 6: 284–292. 21116130 10.4161/epi.6.3.14108 PMC3092676

[62] Wu S, Huang J, Dong J, Pan D (2003) hippo encodes a Ste-20 family protein kinase that restricts cell proliferation and promotes apoptosis in conjunction with salvador and warts. Cell 114: 445–456. 12941273 10.1016/s0092-8674(03)00549-x

[63] Huang J, Wu S, Barrera J, Matthews K, Pan D (2005) The Hippo signaling pathway coordinately regulates cell proliferation and apoptosis by inactivating Yorkie, the Drosophila Homolog of YAP. Cell 122: 421–434. 16096061 10.1016/j.cell.2005.06.007

[64] Zhou D, Conrad C, Xia F, Park JS, Payer B, et al (2009) Mst1 and Mst2 maintain hepatocyte quiescence and suppress hepatocellular carcinoma development through inactivation of the Yap1 oncogene. Cancer Cell 16: 425–438. 19878874 10.1016/j.ccr.2009.09.026 PMC3023165

[65] Polesello C, Huelsmann S, Brown NH, Tapon N (2006) The Drosophila RASSF homolog antagonizes the hippo pathway. Curr Biol 16: 2459–2465. 17174922 10.1016/j.cub.2006.10.060 PMC1828611

[66] Prober DA, Edgar BA (2000) Ras1 promotes cellular growth in the Drosophila wing. Cell 100: 435–446. 10693760 10.1016/s0092-8674(00)80679-0

[67] Donninger H, Vos MD, Clark GJ (2007) The RASSF1A tumor suppressor. J Cell Sci 120: 3163–3172. 17878233 10.1242/jcs.010389

[68] van der Weyden L, Tachibana KK, Gonzalez MA, Adams DJ, Ng BL, et al (2005) The RASSF1A isoform of RASSF1 promotes microtubule stability and suppresses tumorigenesis. Mol Cell Biol 25: 8356–8367. 16135822 10.1128/MCB.25.18.8356-8367.2005 PMC1234312

[69] Tommasi S, Dammann R, Zhang Z, Wang Y, Liu L, et al (2005) Tumor susceptibility of Rassf1a knockout mice. Cancer Res 65: 92–98. 15665283

[70] O'Neill E, Rushworth L, Baccarini M, Kolch W (2004) Role of the kinase MST2 in suppression of apoptosis by the proto-oncogene product Raf-1. Science 306: 2267–2270. 15618521 10.1126/science.1103233

[71] Matallanas D, Romano D, Yee K, Meissl K, Kucerova L, et al (2007) RASSF1A elicits apoptosis through an MST2 pathway directing proapoptotic transcription by the p73 tumor suppressor protein. Mol Cell 27: 962–975. 17889669 10.1016/j.molcel.2007.08.008 PMC2821687

[72] Dumur CI, Dechsukhum C, Ware JL, Cofield SS, Best AM, et al (2003) Genome-wide detection of LOH in prostate cancer using human SNP microarray technology. Genomics 81: 260–269. 12659810 10.1016/s0888-7543(03)00020-x

[73] Eckfeld K, Hesson L, Vos MD, Bieche I, Latif F, et al (2004) RASSF4/AD037 is a potential ras effector/tumor suppressor of the RASSF family. Cancer Res 64: 8688–8693. 15574778 10.1158/0008-5472.CAN-04-2065

[74] Kudo T, Ikeda M, Nishikawa M, Yang Z, Ohno K, et al (2012) The RASSF3 Candidate Tumor Suppressor Induces Apoptosis and G1-S Cell-Cycle Arrest via p53. Cancer Res 72: 2901–2911. 22593196 10.1158/0008-5472.CAN-12-0572

[75] Hesson LB, Cooper WN, Latif F (2007) The role of RASSF1A methylation in cancer. Dis Markers 23: 73–87. 17325427 10.1155/2007/291538 PMC3850810

[76] Irimia M, Fraga MF, Sanchez-Cespedes M, Esteller M (2004) CpG island promoter hypermethylation of the Ras-effector gene NORE1A occurs in the context of a wild-type K-ras in lung cancer. Oncogene 23: 8695–8699. 15378027 10.1038/sj.onc.1207914

[77] Jacquemart IC, Springs AE, Chen WY (2009) Rassf3 is responsible in part for resistance to mammary tumor development in neu transgenic mice. Int J Oncol 34: 517–528. 19148488

[78] Cooper WN, Hesson LB, Matallanas D, Dallol A, von Kriegsheim A, et al (2009) RASSF2 associates with and stabilizes the proapoptotic kinase MST2. Oncogene 28: 2988–2998. 19525978 10.1038/onc.2009.152 PMC2829092

[79] Song H, Oh S, Oh HJ, Lim DS (2010) Role of the tumor suppressor RASSF2 in regulation of MST1 kinase activity. Biochem Biophys Res Commun 391: 969–973. 19962960 10.1016/j.bbrc.2009.11.175

[80] Musteanu M, Blaas L, Zenz R, Svinka J, Hoffmann T, et al (2012) A mouse model to identify cooperating signaling pathways in cancer. Nat Methods 9: 897–900. 22863881 10.1038/nmeth.2130

[81] Matsuda T, Cepko CL (2007) Controlled expression of transgenes introduced by in vivo electroporation. Proc Natl Acad Sci U S A 104: 1027–1032. 17209010 10.1073/pnas.0610155104 PMC1764220

[82] Boussif O, Lezoualc'h F, Zanta MA, Mergny MD, Scherman D, et al (1995) A versatile vector for gene and oligonucleotide transfer into cells in culture and in vivo: polyethylenimine. Proc Natl Acad Sci U S A 92: 7297–7301. 7638184 10.1073/pnas.92.16.7297 PMC41326

[83] DuPage M, Dooley AL, Jacks T (2009) Conditional mouse lung cancer models using adenoviral or lentiviral delivery of Cre recombinase. Nat Protoc 4: 1064–1072. 19561589 10.1038/nprot.2009.95 PMC2757265

[84] Truett GE, Heeger P, Mynatt RL, Truett AA, Walker JA, et al (2000) Preparation of PCR-quality mouse genomic DNA with hot sodium hydroxide and tris (HotSHOT). Biotechniques 29: 52, 54. 10907076 10.2144/00291bm09

