# Supplementary figures and images for: Ras Effector Mutant Expression Suggest a Negative Regulator Inhibits Lung Tumor Formation
Source: PLoS One. 2014 Jan 28;9(1):e84745. doi: 10.1371/journal.pone.0084745 (PMC3904846; doi:10.1371/journal.pone.0084745)

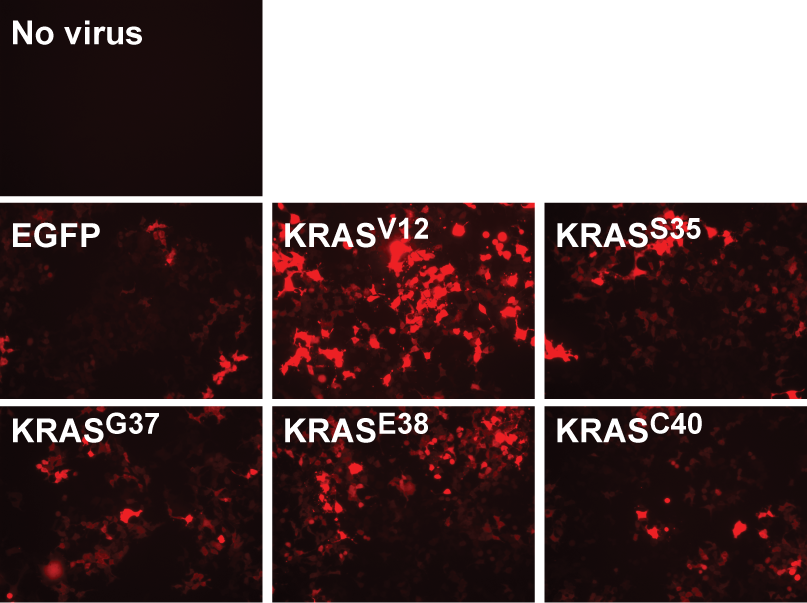

Supplement: Figure S1 — Infection of 293T L9.2 Cre reporter cells that express dsRed upon Cre expression. In each condition, 2.5×10^5^ cells were infected with 6.9×10^6^ IU the same viruses that were used to infect mice. (TIF) [file pone.0084745.s001.tif]

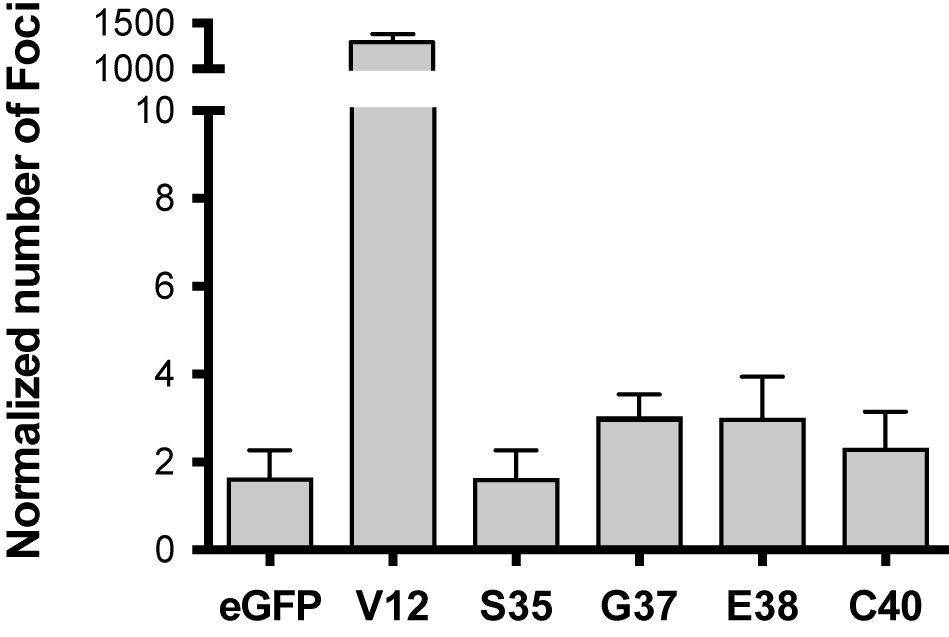

Supplement: Figure S2 — Focus formation in 3T3 cells stably expressing the different KRAS effector domain mutants. 1×10^4^ cells of each stable cell line were seeded with 2.5×10^6^ of the parental 3T3 C5 cell line and left at confluency for 14 days. In the KRASV12 condition, 1×10^3^ cells were seeded instead of 1×10^4^ cells to be able to count single foci and the number was normalized thereafter. Error bars: standard error of the means (SEM). (TIF) [file pone.0084745.s002.tif]

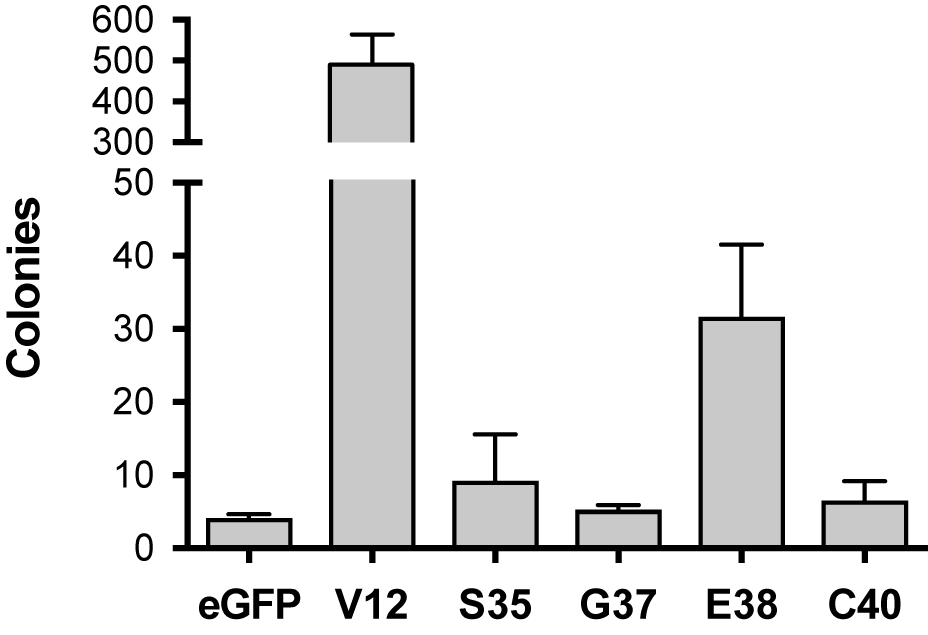

Supplement: Figure S3 — Colony formation in soft agar of 3T3 cell lines expressing the KRAS effector domain mutants. In each well, 1×10^4^ cells were seeded in DMEM with 0.35% low melting point agarose. After 14 days, the number of colonies were stained with MTT and counted. Error bars: SEM. (TIF) [file pone.0084745.s003.tif]

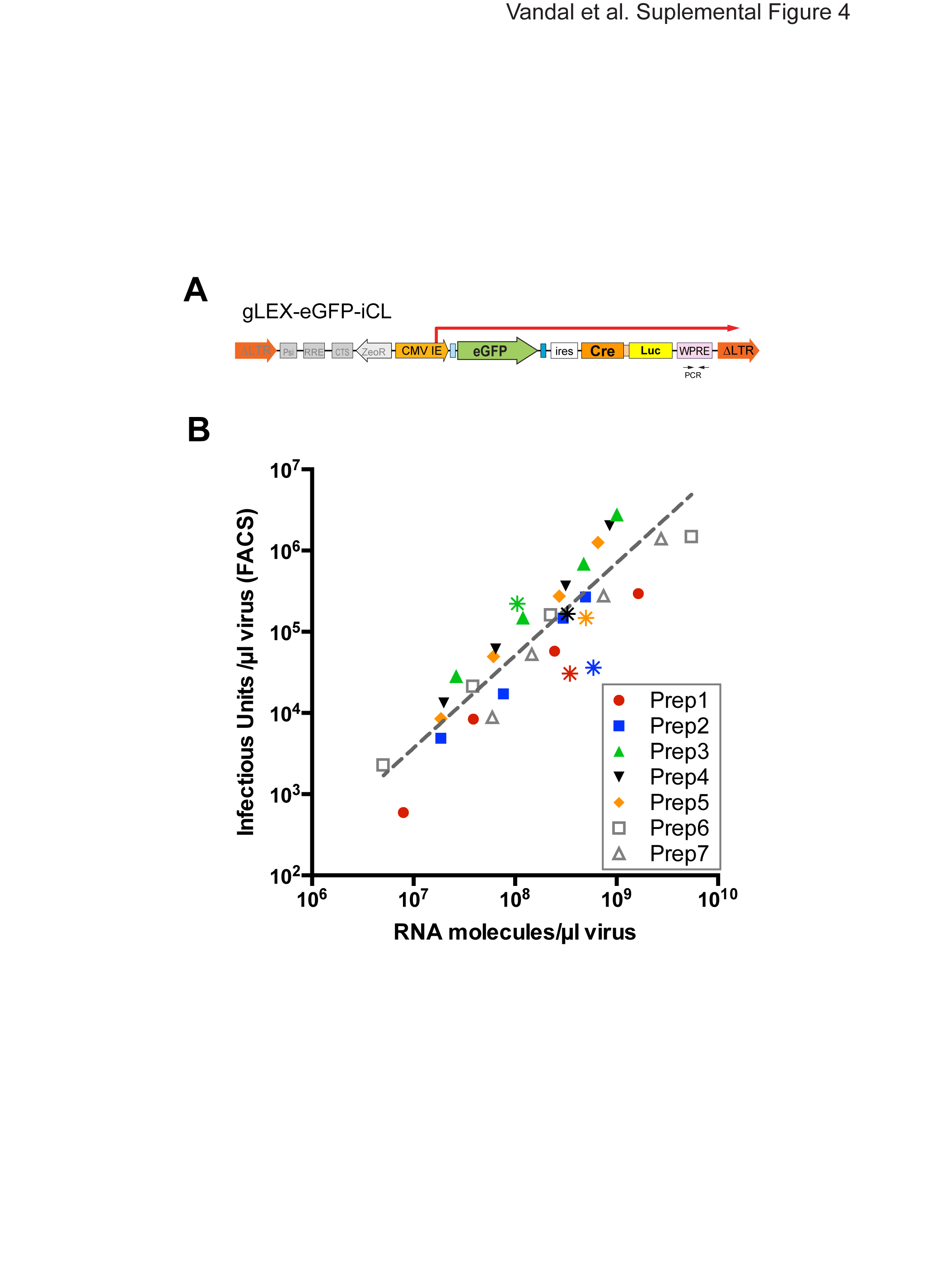

Supplement: Figure S4 — Determining titre of concentrate lentivirus. A) Schematic representation of pLEX-eGFP-ires Cre(2a)Luc (pLEX-eGFP-iCL), a gLEXiCL-derived that expresses eGFP transcriptionally upstream of the Cre(2a)Luc fusion. Indicated is the location of the PCR primers used to quantify lentiviral RNA-derived molecules. B) Seven independent LEX-eGFP-iCL lentiviral preparations were generated and were concentrated, with a small aliquot of preps 1–5 being frozen directly. These viral preps were split, diluted and used to infect 293T cells or to isolate RNA for RT PCR analysis. The graph represents correlation between infectious units, as judged by GFP positivity with FACS analysis, and RNA molecules, by RT PCR. Asterisks indicate the values obtained for undiluted virus and are color-coded with the corresponding prep the figure legend. Preps 6 and 7 were not tested as undiluted viruses. The dotted line indicates the line of best fit the correlation coefficient of r = 0.93. (TIF) [file pone.0084745.s004.tif]

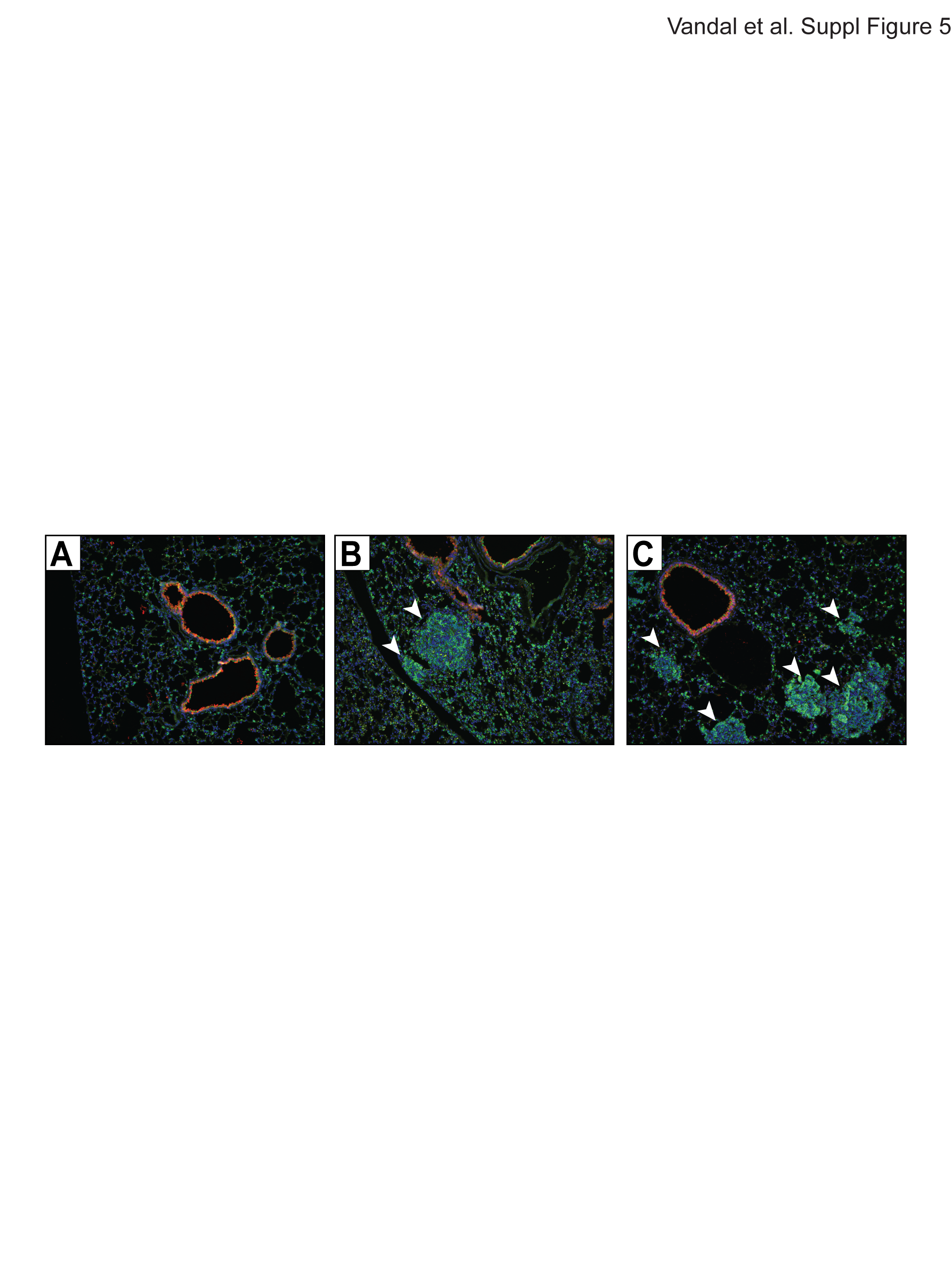

Supplement: Figure S5 — Lentiviral and Adenoviral activation of BrafCA produce similar tumors. A) Braf+/+ or B) BrafCA/+ mice were infected with 108 IU of pLEX-EGFP-iCL lentivirus and lung tissue was obtained 16 weeks postinfection. The tissue of was analysed by immunoflourescence for Clara Cell Antigen (CCA, in red), which marks Clara cells and Surfactant Protein C (SPC, in green), which marks type II pneumocytes. Nuclei are stained blue with DAPI. C) BrafCA/+ mice were infected with 5×10^6^ PFU of Adenoviral Cre and analysed as in B). Note tumors (marked by arrowheads) initiated with either adenovirus or lentivirus stain negative for CCA and positive for SPC. (TIF) [file pone.0084745.s005.tif]

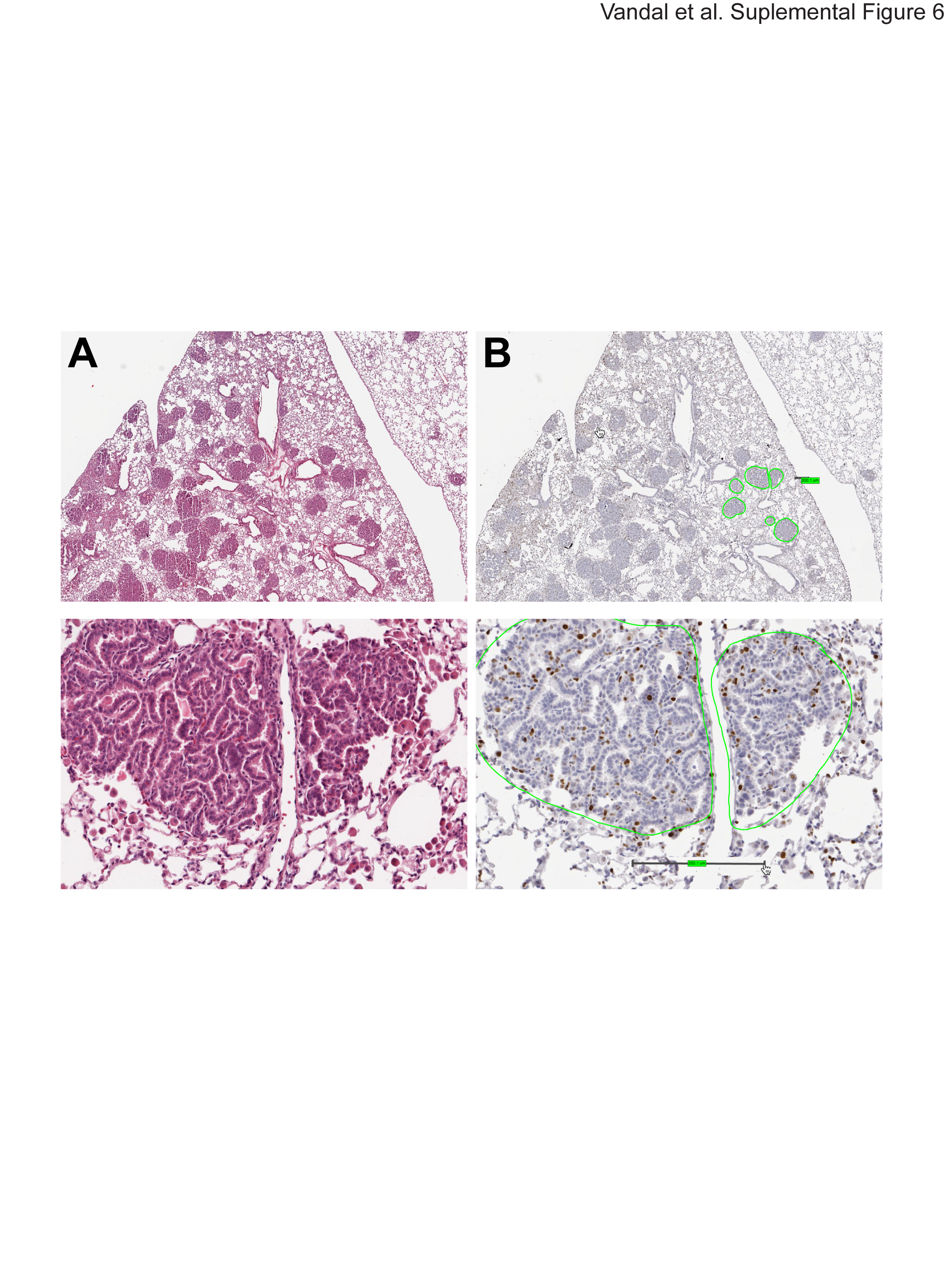

Supplement: Figure S6 — Representative staining for Ki67 determination. Representative images of A) H&E and B) Ki67 stained lung tissues at low (upper) and high (lower) magnification. Aperio software was used to quantify the percentage of Ki67 positive nuclei. Analysis was focused on individual tumours, which were manually were circled (6 tumors are shown circled in green as an example). (TIF) [file pone.0084745.s006.tif]
