# Supplementary material for: Ras Effector Mutant Expression Suggest a Negative Regulator Inhibits Lung Tumor Formation
Source: PLoS One. 2014 Jan 28;9(1):e84745. doi: 10.1371/journal.pone.0084745 (PMC3904846; doi:10.1371/journal.pone.0084745)
Supplement: Methods S1 — (DOCX) [file pone.0084745.s007.docx]

Supplemental file

**Supplemental MATERIAL AND METHODS**

*Protein Expression and Immunoblotting.* Experiments were conducted in 6-well dishes, where 1x105 293T cells were seeded in each well and transfected with 2µg of DNA using polyethylenimine. After 24h, the medium was changed to remove the transfection mix and the cells were rinsed twice with serum-free DMEM to remove any traces of serum. The cells were then incubated for 24h in serum-free DMEM. The cells were then washed twice with PBS and lysed with a 1% Triton X-100 lysis buffer containing 150 mM NaCl, 20 mM HEPES, 10% glycerol, 1mM EDTA, 1mM PMSF, 1µg/ml aprotinin, 1 µg/ml pepstatin, 1 µg/ml leupeptin and 1 mM sodium orthovanadate.

Protein concentration was determined using the Novagen BCA protein assay kit following the manufacturer’s instructions. The proteins of each lysate (30 µg) were mixed in Laemmli sample buffer (67 mM Tris-HCl pH 6.8, 10% glycerol, 1.25% SDS, 0.0025% bromophenol blue and 2.5% β-mercaptoethanol) and separated by SDS-PAGE on a 10% acrylamide gel. Proteins were then transferred on PVDF membranes. The membranes were blocked with 5% non-fat powdered milk in TBST (50 mM Tris pH 7.6, 150 mM NaCL, 0.05% Tween-20). They were then incubated with the primary antibodies overnight at 4°C. The primary antibodies used were ERK1/2 1:1000 (Cell Signalling #9102) and Phospho-ERK1/2 1:1000 (Cell signalling #9106). The membranes were washed 3 times with TBST for 10 minutes each and incubated horseradish peroxydase-coupled secondary antibody for 1 hour at room temperature in the blocking solution. The secondary antibodies used were donkey anti-rabbit 1:5000 (Amersham #NA934) and sheep anti-mouse 1:5000 (Amersham #NA931). The membranes were washed again 3 times with TBST for 10 minutes each. The antibodies were detected using an ECL detection kit (Amersham) and exposed on X-Ray film (Thermo scientific).
