# Supplementary material for: Ras Effector Mutant Expression Suggest a Negative Regulator Inhibits Lung Tumor Formation
Source: PLoS One. 2014 Jan 28;9(1):e84745. doi: 10.1371/journal.pone.0084745 (PMC3904846; doi:10.1371/journal.pone.0084745)
Supplement: Table S1 — (DOCX) [file pone.0084745.s008.docx]

Supplemental Table 1. Tumor penetrance, expressed in average number of tumors observed per lung ± SEM, in *Braf^CA/+^* mice infected by tracheal intubation with 1-2x10^7^ infectious units of indicated lentivirus.

|  | **8weeks** | |  | **16weeks** | |
| --- | --- | --- | --- | --- | --- |
| **Virus** | Mice with tumors | Avg. tumors/lung |  | Mice with tumors | Avg. tumors/lung |
| EGFP | 2/4 | 3.0 ± 2.0 |  | 1/2 | 18.0 |
| KRAS^V12^ | 4/5 | 1.75 ± 0.3 |  | 0/3 | 0 |
| KRAS^V12/S35^ | 1/2 | 3.0 |  | 2/2 | 2.0 |
| KRAS^V12/G37^ | 2/2 | 1.5 ± 0.5 |  | 2/3 | 3.0 |
| KRAS^V12/E38^ | 1/2 | 1.0 |  | 3/3 | 2.3 ± 0.3 |
| KRAS^V12/C40^ | 1/2 | 1.0 |  | 3/3 | 2.0 ± 0.6 |
